# Development and Validation of a Robust and Interpretable Early Triaging Support System for Patients Hospitalized With COVID-19: Predictive Algorithm Modeling and Interpretation Study

**DOI:** 10.2196/52134

**Published:** 2024-01-11

**Authors:** Sangwon Baek, Yeon joo Jeong, Yun-Hyeon Kim, Jin Young Kim, Jin Hwan Kim, Eun Young Kim, Jae-Kwang Lim, Jungok Kim, Zero Kim, Kyunga Kim, Myung Jin Chung

**Affiliations:** 1 Medical AI Research Center Samsung Medical Center Seoul Republic of Korea; 2 Center for Data Science New York University New York, NY United States; 3 Department of Radiology, Research Institute for Convergence of Biomedical Science and Technology Pusan National University Yangsan Hospital Yangsan Republic of Korea; 4 Department of Radiology Chonnam National University Hospital Gwangju Republic of Korea; 5 Department of Radiology Keimyung University Dongsan Hospital Daegu Republic of Korea; 6 Department of Radiology Chungnam National University Hospital Daejeon Republic of Korea; 7 Department of Radiology Gachon University Gil Medical Center Incheon Republic of Korea; 8 Department of Radiology, School of Medicine Kyungpook National University Daegu Republic of Korea; 9 Department of Infectious Diseases Chungnam National University Sejong Hospital Sejong Republic of Korea; 10 Department of Data Convergence & Future Medicine Sungkyunkwan University School of Medicine Seoul Republic of Korea; 11 Biomedical Statistics Center, Research Institute for Future Medicine Samsung Medical Center Seoul Republic of Korea; 12 Department of Digital Health SAIHST, Sungkyunkwan University Seoul Republic of Korea; 13 Department of Radiology Samsung Medical Center Seoul Republic of Korea

**Keywords:** COVID-19, prognosis, prognostic, prognostics, prediction model, early triaging, interpretability, machine learning, predict, prediction, predictive, triage, triaging, emergency, severity, biomarker, biomarkers, SHAP, Shapley, clustering, hospital admission, hospital admissions, hospitalize, hospitalization, hospitalizations, neural network, neural networks, deep learning, Omicron, SARS-CoV-2, coronavirus

## Abstract

**Background:**

Robust and accurate prediction of severity for patients with COVID-19 is crucial for patient triaging decisions. Many proposed models were prone to either high bias risk or low-to-moderate discrimination. Some also suffered from a lack of clinical interpretability and were developed based on early pandemic period data. Hence, there has been a compelling need for advancements in prediction models for better clinical applicability.

**Objective:**

The primary objective of this study was to develop and validate a machine learning–based Robust and Interpretable Early Triaging Support (RIETS) system that predicts severity progression (involving any of the following events: intensive care unit admission, in-hospital death, mechanical ventilation required, or extracorporeal membrane oxygenation required) within 15 days upon hospitalization based on routinely available clinical and laboratory biomarkers.

**Methods:**

We included data from 5945 hospitalized patients with COVID-19 from 19 hospitals in South Korea collected between January 2020 and August 2022. For model development and external validation, the whole data set was partitioned into 2 independent cohorts by stratified random cluster sampling according to hospital type (general and tertiary care) and geographical location (metropolitan and nonmetropolitan). Machine learning models were trained and internally validated through a cross-validation technique on the development cohort. They were externally validated using a bootstrapped sampling technique on the external validation cohort. The best-performing model was selected primarily based on the area under the receiver operating characteristic curve (AUROC), and its robustness was evaluated using bias risk assessment. For model interpretability, we used Shapley and patient clustering methods.

**Results:**

Our final model, RIETS, was developed based on a deep neural network of 11 clinical and laboratory biomarkers that are readily available within the first day of hospitalization. The features predictive of severity included lactate dehydrogenase, age, absolute lymphocyte count, dyspnea, respiratory rate, diabetes mellitus, c-reactive protein, absolute neutrophil count, platelet count, white blood cell count, and saturation of peripheral oxygen. RIETS demonstrated excellent discrimination (AUROC=0.937; 95% CI 0.935-0.938) with high calibration (integrated calibration index=0.041), satisfied all the criteria of low bias risk in a risk assessment tool, and provided detailed interpretations of model parameters and patient clusters. In addition, RIETS showed potential for transportability across variant periods with its sustainable prediction on Omicron cases (AUROC=0.903, 95% CI 0.897-0.910).

**Conclusions:**

RIETS was developed and validated to assist early triaging by promptly predicting the severity of hospitalized patients with COVID-19. Its high performance with low bias risk ensures considerably reliable prediction. The use of a nationwide multicenter cohort in the model development and validation implicates generalizability. The use of routinely collected features may enable wide adaptability. Interpretations of model parameters and patients can promote clinical applicability. Together, we anticipate that RIETS will facilitate the patient triaging workflow and efficient resource allocation when incorporated into a routine clinical practice.

## Introduction

During the COVID-19 pandemic, the global health care system confronted an urgent threat despite concerted efforts from health care institutions and providers to contain the rapid spread of the disease, which has claimed the lives of 6.97 million people as of October 2023 [1]. The overwhelming influx of patients into hospitals strained medical resources and hindered optimal treatment provision by health care practitioners [2]. This global outbreak may continue to exist with the advent of new SARS-CoV-2 variants due to its tendency to mutate during host adaptation [3]. Recently, a high proportion of population immunity and decreasing fatality rates initiated global movements toward endemic status following the World Health Organization’s (WHO) announcement that COVID-19 is no longer a public health emergency of international concern [4,5]. However, COVID-19 cases continue to rise with the emergence of new subvariants, such as SARS-CoV-2 EG.5 and BA.2.86 [6,7]. Therefore, a robust and interpretable early triaging system is necessary to accurately triage patients in preparation for the next pandemic [8].

Many prognostic models for patients with COVID-19 severity and mortality have been proposed, yet most were reported unsuitable for clinical application by several systematic review studies [9-11]. Most models were either at a high or unclear risk of bias (Wynants et al [10]: 305 out of 310 studies, 98.4%; Buttia et al [11]: 312 out of 314 studies, 99.4%) such that their reported discriminative performances were deemed neither reliable nor generalizable [10,11]. These high-risk models were developed with predictors selected based on univariable analysis, failed to deal with model overfitting represented by miscalibration, performed no or limited external validations with sufficient samples, imputed missing data without a clear explanation, or considered a limited number of machine learning (ML) algorithms [10,11]. Although there were some models with a low risk of bias, these models had low to moderate discriminative power, were based on the data from the early pandemic period, and had limited clinical interpretability [9,10]. Therefore, the development of a robust, interpretable, and generalizable model with high discriminative power is required to provide practical benefit in managing the next possible pandemic [12,13].

We aimed to develop and validate an ML-based Robust and Interpretable Early Triaging Support (RIETS) system to predict severity based on routinely collected biomarkers using a nationwide multicenter cohort. In addition, we tried to improve model interpretability through patient clustering and characterization.

## Methods

### Ethical Considerations

The study protocol was approved and the requirement for informed consent was waived by the institutional review boards (IRBs) of all participating hospitals. In addition, the use and management of a cloud-based data storage platform for the secondary analysis was approved by the IRB of Samsung Medical Center (SMC 2020-09-100-002). All unique identifiers were removed prior to uploading. All data in the study database were assigned a research specific serial number and deidentified to protect the confidentiality of the study patients.

### Study Setting and Design

**Figure 1:**
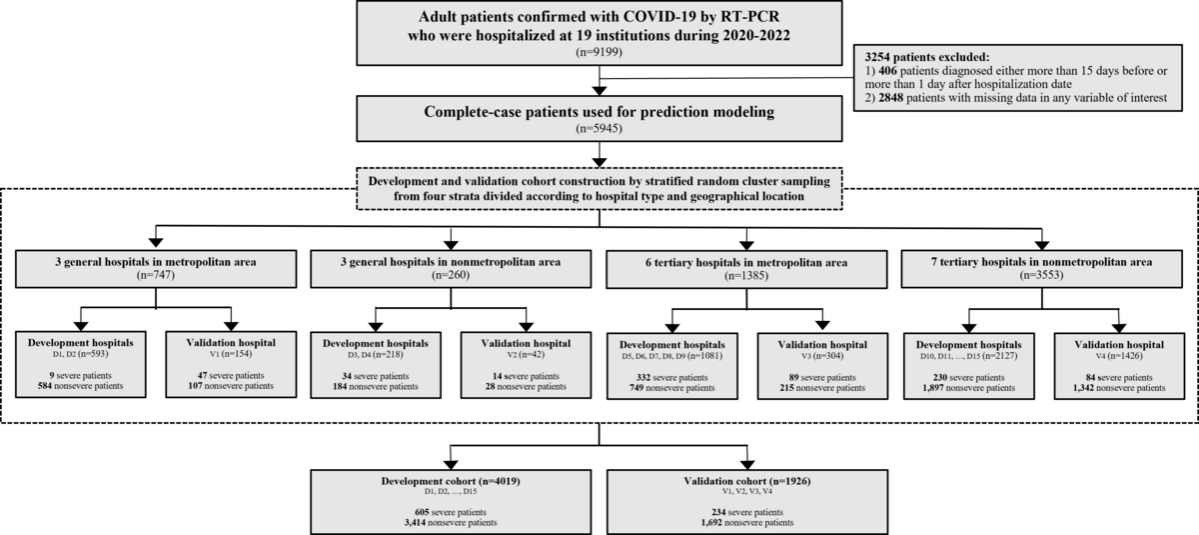
Patient flowchart depicting the generation of development and validation cohorts among hospitalized patients with COVID-19 (n=5945). Stratified random cluster sampling was applied to segment the cohorts based on hospital type (general vs tertiary) and geographical location (metropolitan vs nonmetropolitan). RT-PCR: real time polymerase chain reaction.

This study was a nationwide, multicenter, retrospective, prognostic study conducted in South Korea. We collected data for adult patients who were confirmed to have COVID-19 via real-time polymerase chain reaction and were hospitalized at 19 main referral hospitals between January 5, 2020, and August 29, 2022 (Methods S1 in Multimedia Appendix 1). Among 9199 hospitalized patients with COVID-19, we excluded 406 patients diagnosed either more than 15 days before or more than 1 day after the hospitalization date, as well as 2848 patients who had missing data in any variable of interest (Figure 1). A total of 5945 patients (5106 nonsevere and 839 severe) remained for the analysis. The 19 hospitals were divided into 4 strata according to hospital type and location: metropolitan area general hospitals, nonmetropolitan area general hospitals, metropolitan area tertiary care hospitals, and nonmetropolitan area tertiary care hospitals. We then used a random cluster sampling method to partition hospitals in each stratum and construct development and validation cohorts. For reporting and bias-risk assessment, we adhered to the following guidelines: Guidelines for Developing Machine Learning Predictive Models in Biomedical Research [14], Transparent Reporting of a multivariable prediction model for Individual Prognosis (TRIPOD; File S1 in Multimedia Appendix 1), and Diagnosis and Prediction Model Risk of Bias Assessment Tool (PROBAST; File S2 in Multimedia Appendix 1) [15].

### Data Collection

A set of data collection guidelines were predetermined by our clinical experts. We developed a standard data collection form and prepared cloud database storage. Adhering to the set guidelines, researchers affiliated with each participating hospital gathered patient data with 32 features from demographic, clinical, laboratory, and radiological findings within the first day of hospitalization. We specified these features based on previous prognostic models and a literature review describing common biomarkers associated with severe COVID-19 [16]. The final severity status of each patient was determined on day 15 of hospitalization. All data collected in each hospital were deidentified and uploaded onto the cloud database storage. The entire data set underwent a quality assurance process, including typo rectification, outlier handling, and double-checking with the electronic health records in each participating hospital.

### Definition of COVID-19 Severity

We declared the COVID-19 severity for patients under one or more of the following conditions during their hospitalization: (1) mechanical ventilation required; (2) extracorporeal membrane oxygenation required; (3) admission to intensive care unit; or (4) patient’s death. This criterion aligns closely with severe status (score of 6 or higher) in the WHO Clinical Progression Scale, which is developed by reaching a consensus among a group of international medical experts [17].

### Identification of Candidate Feature Subsets

**Figure 2:**
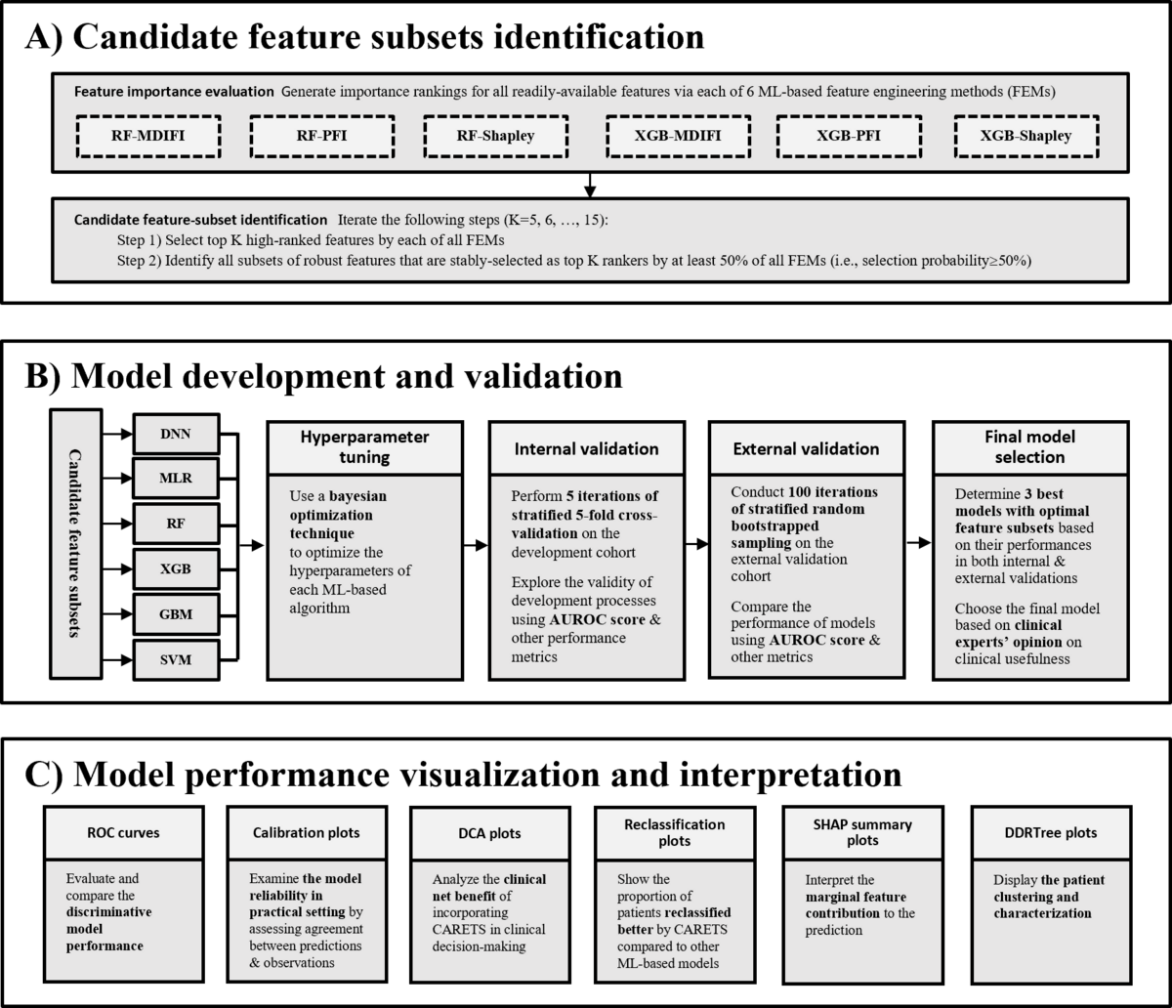
Machine learning–based pipeline for developing and validating the prognosis prediction model for COVID-19 severity. AUROC: area under receiver operating characteristic curve; DCA: decision curve analysis; DDRTree: Discriminative dimensionality reduction by learning a tree; DNN: deep neural network; GBM: gradient boosting machine; MLR: multivariable logistic regression; RF: random forest; RF-MDIFI: random forest–based mean decrease in Gini Impurity feature importance method; RF-PFI: random forest–based permutation feature importance method; RF-Shapley: random forest–based Shapley method; ROC: receiver operating characteristic curve; SHAP: Shapley additive explanations; SVM: support vector machine; XGB: extreme gradient boosting; XGB-BFI: extreme gradient boosting–based built-in feature importance method; XGB-PFI: extreme gradient boosting–based permutation feature importance method; XGB-Shapley: extreme gradient boosting–based Shapley method.

Among the 32 collected features, 27 readily accessible features without missing data remained for prediction modeling (Figure 2A). In order to identify subsets of robust features against feature selection methods, we considered 6 feature engineering methods (FEMs) based on 2 ML algorithms with optimal hyperparameter tuning and 4 feature importance measures: random forest (RF)-based mean decreases in Gini impurity feature importance; RF-based permutation feature importance; RF-based Shapley values; extreme gradient boosting (XGB)-based built-in feature importance; XGB-based permutation feature importance; and XGB-based Shapley values.

All features were ranked by importance measure by each FEM. We considered various criteria (ie, top K; K=5, 6, …, 15) for high-ranking features, termed high rankers. Based on each criterion K, we generated candidate feature subsets in two steps: (1) we selected high rankers with K highest importance rankings by each FEM, and (2) we identified features that were stably selected as high rankers by at least 50% of all FEMs. This process resulted in candidate subsets of robust features. The set of all 27 features was used as the reference model to identify the performance improvements in subset models during model evaluation.

### Model Development and Validation

A total of 60 candidate feature subsets were used for the model development, including 59 identified subsets of robust features and the reference set of all 27 features (Figure S1 in Multimedia Appendix 1). We first fine-tuned the hyperparameters of 6 ML-based algorithms, namely, deep neural network (DNN), multivariable logistic regression, RF, XGB, gradient boosting machine, and support vector machine, by applying Bayesian optimization on the development cohort. Then, we simultaneously developed all possible 360 combinations of 6 ML-based algorithms and those 60 feature subsets and evaluated the performances in both internal and external validations (Figure 2B). The model predictive performance was evaluated using the area under the receiver operating characteristic curve (AUROC) score and other cutoff-based measures, such as sensitivity, specificity, positive and negative predicted values, positive and negative likelihood ratios, and diagnostic odds ratio.

Model development and internal validation were done with the development cohort in the following steps. First, we used 5 iterations of stratified 5-fold cross-validation to explore the internal validity of each combination of feature subsets and ML algorithms as a model development procedure. The procedures were evaluated by the mean values of performance metrics and their 95% CIs calculated from the repeated cross-validation process (Methods S2 in Multimedia Appendix 1). Second, we used the entire development cohort to construct prediction models based on each of the internally validated development procedures.

External validation was conducted for the prediction models with the validation cohort. Each prediction model was evaluated by the mean values and 95% CIs of performance measures that were calculated from 100 iterations of bootstrapped sampling (Methods S2 in Multimedia Appendix 1). The final prediction model was proposed in three steps: (1) for each ML algorithm, we selected the optimal feature subset that produced the model with best discriminative performance in both internal and external validations; (2) the 3 prediction models with the best predictivity were chosen to compare their usefulness via calibration, reclassification improvement, and decision curve analysis (DCA); (3) the DNN-based final prediction model, RIETS, was proposed by considering its discriminative ability along with clinical applicability [18].

### Model Performance Visualization and Feature Interpretation

We used graphical representations to visualize the performance of RIETS contrasted with other ML-based models and provided interpretation for the selected features (Figure 2C; Methods S3 in Multimedia Appendix 1). Receiver operating characteristic curves demonstrated the discriminative model performance. Calibration plots implicated the model’s reliability in practical settings by displaying the correlation between predicted and observed risks. DCA plots indicated the net benefit of incorporating the model in clinical decision-making by quantifying the weighted trade-off between true positive and false positive identifications [19]. Reclassification plots displayed the proportion of patients that were reclassified correctly or incorrectly by RIETS compared to other ML-based models. Lastly, the Shapley additive explanations summary plot interpreted the contributions of individual features in RIETS when classifying severe and nonsevere cases [20].

### Patient Clustering and Characterization Using Discriminative Dimensionality Reduction

We used discriminative dimensionality reduction via learning a tree (DDRTree) to cluster and characterize patients based on the features in RIETS. DDRTree is a tree-based unsupervised learning technique that reduces multidimensional features into a 2-dimensional space to visualize patients in the form of a tree structure (see Methods S3 in Multimedia Appendix 1 for procedures). This tool is known to capture cluster information with higher accuracy compared to conventional dimensionality reduction methods [21,22].

**Figure 3:**
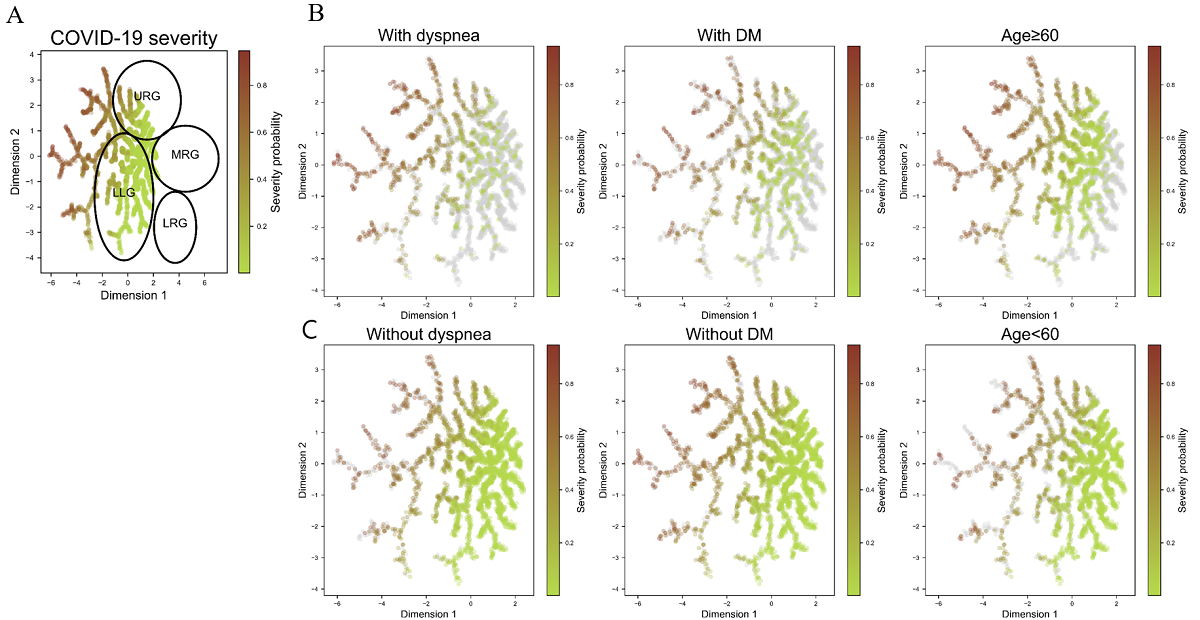
Patient clustering based on features in RIETS and characterization with dyspnea, DM, age, and severity. (A) DDRTree plot for severity probability. (B) DDRTree plot for patients with dyspnea, DM, and age ≥60 years. (C) DDRTree plot for patients with dyspnea, DM, and age <60 years. Points closer to dark red indicate a high severity probability, while points closer to light green indicate a low severity probability. DDRTree: discriminative dimensionality reduction via learning a tree; DM: diabetes mellitus; LLG: lower left group; LRG: lower right group; MRG: middle right group; RIETS: robust and interpretable early triaging support system; URG: upper right group.

**Figure 4:**
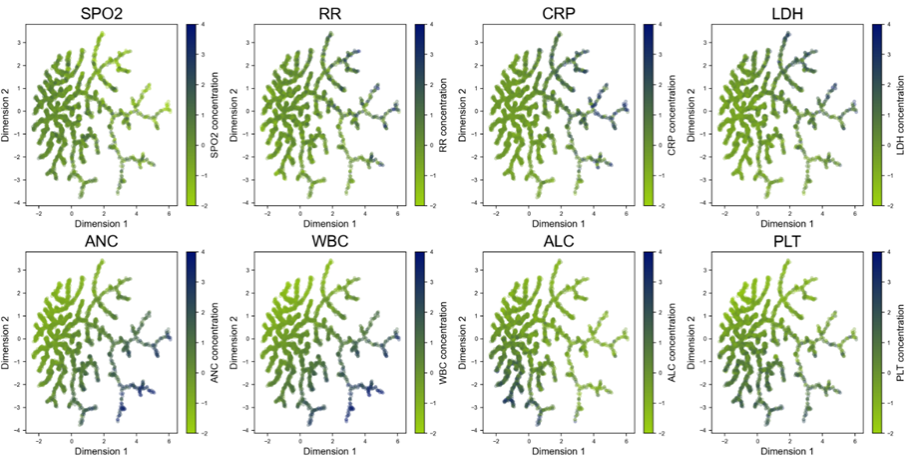
Patient clustering based on features in RIETS and characterization with vital signs and laboratory results. A dark blue color indicates a high concentration and a light green color indicates a low concentration of each corresponding feature. ALC: absolute lymphocyte count; ANC: absolute neutrophil count; CRP: c-reactive protein; DDRTree: discriminative dimensionality reduction via learning a tree; LDH: lactate dehydrogenase; PLT: platelet count; RIETS: Robust and Interpretable Early Triaging Support; RR: respiratory rate; SPO2: saturation of peripheral oxygen; WBC: white blood cell.

Each patient in a tree was colored with dark red to indicate high odds for severity and light green to indicate low odds for severity (Figure 3). Then, in Figure 4, dark blue and light green colors were overlaid to represent high and low concentrations of each laboratory marker, respectively. Severity risk of each patient can be identified through Figure 3A, the risk distribution with or without a pre-existing condition can be seen in Figures 3B and 3C, and feature values associated with each patient can be inferred from Figure 4. We integrated these observations to cluster patients into subgroups and characterize each subgroup (subgroup boundaries are shown in Figure 3A).

### Definition of Variant-Dominant Periods

The predominant circulating variant at the time of hospitalization was identified through viral whole genome sequencing and could differ across nations [23]. According to predominant circulating variants during the pandemic in South Korea, we segmented our study period into 3 variant-dominant periods and constructed the corresponding patient subcohorts: original Alpha-dominant period (January 5, 2020 to May 1, 2021), Delta-dominant period (May 1, 2021 to November 24, 2021), and Omicron-dominant period (November 24, 2021 to August 24, 2022) [23].

### Analysis of Model Transportability on Omicron Variant Cases

We developed modifications of RIETS to explore its prediction transportability across different variant-dominant periods. Each modified model was constructed using the variant dominant subcohorts in the development cohort. For instance, the “RIETS-All” model was based on the entire development cohort and the “RIETS-Omicron” model was based on the Omicron-dominant development cohort. We evaluated all possible combinations of modified RIETS and compared their discriminative performances among patients in the external validation cohort. Consequently, we identified the best-performing model, named “RIETS-Ensemble,” that integrates the 3 models based on original Alpha-Omicron, Delta-Omicron, and Omicron. Then, the “RIETS-Ensemble” model was contrasted to the “RIETS-All” and “RIETS-Omicron” models to visualize marginal improvements. All developed models were compared using the AUROC as a measure for discriminative performance.

### Statistical Analysis

Patient characteristics were summarized as median (IQR) and number (%) for continuous and categorical variables, respectively, and compared between the development and validation cohorts via absolute standardized mean difference (ASMD). The ASMD was calculated using Cohen D and H formulas for continuous and categorical variables, respectively. No considerable difference was identified with an ASMD below 0.2. For cutoff-based performance measures, Youden index was used to find an optimal threshold at which the average of sensitivity and specificity was maximized. The integrated calibration index (ICI), derived from the weighted mean difference between observed and predicted probabilities for the outcome, was used to quantify and assess calibration. ICI was preferred over other calibration metrics (eg, calibration-in-the-large and slope) due to its high stability from capturing the entire range of predicted probabilities during its computation [24]. A 2-sided *P* value below 0.05 was set to declare statistical significance. All statistical analyses were performed using Python (Python Software Foundation, version 3.9).

## Results

### Patient Characteristics

Among the 5945 hospitalized patients with COVID-19 used for the development and validation of RIETS, 4019 (67.6%) and 1926 (32.4%) were allocated into the development and validation cohorts, respectively (Table 1). The median age was higher in the development cohort than in the validation cohort (mean 60, SD 45-70 years vs mean 55, SD 35-65 years, respectively; ASMD=0.333). The proportion of male patients was similar in both the development (n=2130, 48.8%) and validation (n=757, 47.9%) cohorts. Hypertension was the most prevalent comorbidity for both the development (n=1622, 37.2%) and validation (n=492, 31.1%) cohorts. While the most frequent symptoms across both cohorts were cough (n=2623, 44.1%) and fever (n=2366, 39.8%), the rankings of observed symptoms were similar in both cohorts. All variables pertaining to vital signs and blood biochemistry showed no considerable difference between cohorts (ASMD<0.2), except for the absolute neutrophil count (ANC; ASMD=0.319).

**Table 1:** Baseline characteristics in the development and validation cohorts of hospitalized South Korean patients with COVID-19.

			Total cohort (n=5945)		Development cohort (n=4019)		Validation cohort (n=1926)		ASMD^a^
**Patient Characteristics**									
	Age (years), median (IQR)	60 (40-70)		60 (45-70)		50 (35-65)		0.333	
	Male sex, n (%)	2887 (48.6)		2130 (48.8)		757 (47.9)		0.018	
**Comorbidities, n (%)**									
	Hypertension	2114 (35.6)		1622 (37.2)		492 (31.1)		0.127	
	Diabetes mellitus	1249 (21)		978 (22.4)		271 (17.2)		0.132	
	Cardiovascular disease	508 (8.5)		389 (8.9)		119 (7.5)		0.050	
	Cancer	477 (8)		369 (8.5)		108 (6.8)		0.061	
	Others	2242 (37.7)		1813 (41.5)		429 (27.2)		0.304	
**Clinical symptoms, n (%)**									
	Fever	2366 (39.8)		1678 (38.4)		688 (43.5)		0.104	
	Cough	2623 (44.1)		1852 (42.4)		771 (48.8)		0.128	
	Sputum	1502 (25.3)		1038 (23.8)		464 (29.4)		0.127	
	Dyspnea	1318 (22.2)		1042 (23.9)		276 (17.5)		0.159	
	Myalgia	1388 (23.3)		938 (21.5)		450 (28.5)		0.162	
	Sore throat	1142 (19.2)		763 (17.5)		379 (24)		0.161	
	Loss of sensor	330 (5.6)		212 (4.9)		118 (7.5)		0.109	
	Gastrointestinal symptom	472 (7.9)		295 (6.8)		177 (11.2)		0.157	
**Vital sign, median (IQR)**									
	Body temperature (℃)	36.6 (36.3-37.2)		36.6 (36.3-37.2)		36.5 (36.3-37.2)		0.051	
	Systolic blood pressure (mmHg)	129 (116-141)		129 (116-140)		129 (117-141)		0.021	
	Diastolic blood pressure (mmHg)	80 (70-87)		80 (70-86)		80 (72-90)		0.200	
	Pulse rate (counts)	84 (74-95)		84 (74-95)		86 (76-97)		0.128	
	Respiratory rate (counts)	20 (18-20)		20 (18-20)		20 (18-20)		0.191	
	SPO2^b^ (%)	97 (96-98)		97 (96-98)		97 (96-98)		0.021	
**Blood biochemistry, median (IQR)**									
	White blood cells (10^3^/µL)	5.2 (4.1-6.9)		5.3 (4.1-7.1)		5.1 (4.0-6.6)		0.143	
	Absolute neutrophil count (10^3^/µL)	3.5 (2.4-5.4)		3.7 (2.5-5.8)		3.0 (2.1-4.3)		0.319	
	Absolute lymphocyte count (10^3^/µL)	1.2 (0.8-1.7)		1.1 (0.8-1.6)		1.3 (1.0-1.8)		0.105	
	Platelet count (10^3^/µL)	200 (157-248)		198 (154-248)		205 (166-248)		0.068	
	C-reactive protein (mg/dL)	1.2 (0.3-5.2)		1.5 (0.3-6.0)		0.7 (0.2-2.9)		0.077	
	Lactate dehydrogenase (U/L)	316 (221-445)		287 (212-428)		369 (289-476)		0.161	

^a^ASMD: absolute standardized mean difference.

^b^SPO2: saturation of peripheral oxygen.

The baseline characteristics were also compared between 839 (14.1%) patients with nonsevere COVID-19 and 5106 (85.9%) patients with severe COVID-19 (Table S1 in Multimedia Appendix 1). Patients with severe COVID-19 were older (mean 70, SD 60-75 years vs mean 55, SD 40-70 years; ASMD=0.733). A larger proportion of patients with severe COVID-19 had dyspnea (ASMD=0.902) and diabetes mellitus (DM; ASMD=0.524). Patients with severe COVID-19 were more likely to have an increased respiratory rate (RR; ASMD=0.911) and decreased saturation of peripheral oxygen (SPO2; ASMD=0.705) upon hospital admission. In addition, patients with severe COVID-19 presented with higher ANC (ASMD=0.971), higher lactate dehydrogenase (LDH; ASMD=0.726), and higher white blood cell (WBC) count (ASMD=0.693).

### Performance of RIETS

**Figure 5:**
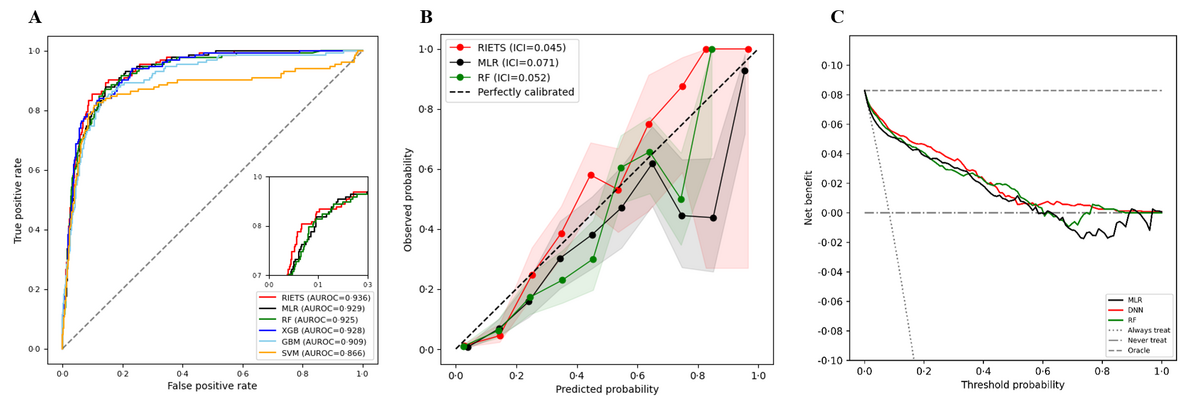
Performance comparisons of RIETS to other prediction models in the external validation. (A) Receiver operating characteristic curves for displaying the discriminative performances. (B) Calibration plots for showing the practical reliability of risk prediction. (C) Decision curve analysis plots for demonstrating the net clinical utility when deployed in a clinical practice. All dashed lines represent the references. Shaded areas represent the 95% CI bands. AUROC: area under receiver operating characteristic curve; GBM: gradient boosting machine; ICI: integrated calibration index; MLR: multivariable logistic regression; RIETS: Robust and Interpretable Early Triaging System; RF: random forest; SVM: support vector machine; XGB: extreme gradient boosting.

RIETS is a DNN-based final model with the subset of 11 features that demonstrated the highest discriminative power (AUROC=0.937, 95% CI 0.935-0.938; diagnostic odds ratio=46.14, 95% CI 43.40-48.87; specificity=0.867, 95% CI 0.865-0.869; sensitivity=0.869, 95% CI 0.864-0.875) amongst the 6 ML-based models (AUROC=0.862-0.929) (Figure 5A and Table 2). RIETS also exhibited a superior discriminative ability compared to the existing low risk of bias models (AUROC=0.60-0.80; Table S2 in Multimedia Appendix 1).

**Table 2:** Discriminative performance of machine learning–based models based on 11 clinical and laboratory features collected within the first day of hospitalization in the internal and external validation (IV and EV, respectively). The IV and EV results were computed from 5 iterations of stratified 5-fold cross-validations and 100 iterations of bootstrapped sampling, respectively. The Youden index was used to determine the optimal cutoff point.

Model and validation type			Predictive measures (95% CI)																	Cutoff
			AUROC^a^		Sensitivity		Specificity		PPV^b^		NPV^c^		LRP^d^		LRN^e^		DOR^f^			
**RIETS^g^**																				0.171
	IV	0.891 (0.889-0.892)		0.852 (0.844-0.860)		0.808 (0.796-0.819)		46.58 (45.24-47.93)		96.59 (96.45-96.73)		4.57 (4.30-4.84)		0.18 (0.17-0.19)		25.96 (24.22-27.71)				
	EV	0.937 (0.935-0.938)		0.869 (0.864-0.875)		0.867 (0.865-0.869)		37.65 (37.07-38.24)		98.63 (98.57-98.69)		6.56 (6.46-6.66)		0.15 (0.14-0.16)		46.14 (43.40-48.87)				
**Multivariable logistic regression**																				0.164
	IV	0.887 (0.886-0.888)		0.847 (0.845-0.849)		0.792 (0.789-0.795)		44.40 (43.99-44.81)		96.40 (96.35-96.45)		4.17 (4.08-4.26)		0.19 (0.19-0.20)		21.93 (21.35-22.52)				
	EV	0.929 (0.927-0.930)		0.879 (0.874-0.884)		0.832 (0.830-0.834)		32.61 (32.08-33.14)		98.67 (98.61-98.73)		5.25 (5.18-5.32)		0.15 (0.14-0.15)		38.10 (36.11-40.10)				
**Random forest**																				0.193
	IV	0.894 (0.893-0.896)		0.848 (0.831-0.866)		0.807 (0.791-0.823)		46.47 (44.98-47.96)		96.51 (96.17-96.85)		4.55 (4.27-4.82)		0.19 (0.17-0.21)		25.40 (23.75-27.05)				
	EV	0.925 (0.923-0.927)		0.863 (0.857-0.869)		0.864 (0.862-0.866)		36.94 (36.30-37.57)		98.56 (98.49-98.62)		6.36 (6.26-6.46)		0.16 (0.15-0.17)		42.35 (40.09-44.60)				
**Extreme gradient boosting**																				0.105
	IV	0.878 (0.875-0.880)		0.833 (0.813-0.853)		0.784 (0.772-0.796)		43.34 (42.36-44.33)		96.08 (95.68-96.47)		4.01 (3.82-4.20)		0.21 (0.19-0.23)		19.54 (18.05-21.02)				
	EV	0.900 (0.898-0.903)		0.826 (0.820-0.832)		0.836 (0.835-0.838)		31.85 (31.33-32.37)		98.11 (98.04-98.19)		5.07 (5.00-5.14)		0.21 (0.20-0.21)		25.39 (24.21-26.57)				
**Gradient boosting machine**																				0.010
	IV	0.879 (0.877-0.882)		0.837 (0.820-0.853)		0.791 (0.775-0.808)		44.66 (42.94-46.37)		96.23 (95.94-96.51)		4.27 (3.95-4.60)		0.20 (0.19-0.22)		21.74 (20.75-22.74)				
	EV	0.907 (0.904-0.910)		0.852 (0.847-0.858)		0.841 (0.839-0.843)		33.15 (32.56-33.74)		98.40 (98.33-98.47)		5.38 (5.30-5.47)		0.18 (0.17-0.18)		32.28 (30.58-33.97)				
**Support vector machine**																				0.105
	IV	0.834 (0.830-0.837)		0.782 (0.771-0.794)		0.833 (0.826-0.841)		48.15 (47.20-49.09)		95.20 (95.00-95.40)		4.88 (4.69-5.08)		0.26 (0.25-0.27)		19.19 (17.97-20.42)				
	EV	0.862 (0.858-0.866)		0.794 (0.787-0.801)		0.899 (0.898-0.901)		42.12 (41.49-42.76)		97.93 (97.85-98.00)		7.92 (7.78-8.05)		0.23 (0.22-0.24)		35.83 (34.22-37.43)				

^a^AUROC: area under receiver operating characteristic curve.

^b^PPV: positive predictive value.

^c^NPV: negative predictive value.

^d^LRP: likelihood ratio positive.

^e^LRN: likelihood ratio negative.

^f^DOR: diagnostic odds ratio.

^g^RIETS: Robust and Interpretable Early Triaging System.

In comparison with other ML-based models, RIETS exhibited net reclassification improvement (0.54%-6.14%), especially on nonsevere cases (2.14%-6.14%) (Table S3 and Figure S2 in Multimedia Appendix 1) and had the most stable prediction during cost sensitivity learning [25] (Figure S3 in Multimedia Appendix 1). RIETS also maintained sustainable prediction transportability (AUROC=0.903, 95% CI 0.897-0.910) on the limited number of cases (n=449, 7.6%) in the Omicron-dominant period when an ensemble learning technique was applied (Figure S4, Table S4, and Methods S4 in Multimedia Appendix 1).

Moreover, a PROBAST evaluation indicated that RIETS has a low risk of bias and minimal concerns regarding applicability (Methods S2 in Multimedia Appendix 1). RIETS also attained the best calibration (ICI=0.041) among comparable ML-based models (ICI=0.052-0.071; Figure 5B). Overall, it showed a higher net clinical benefit than the “intervention for none” and “intervention for all” reference strategies in DCA (Figure 5C).

### Feature Interpretation

**Figure 6:**
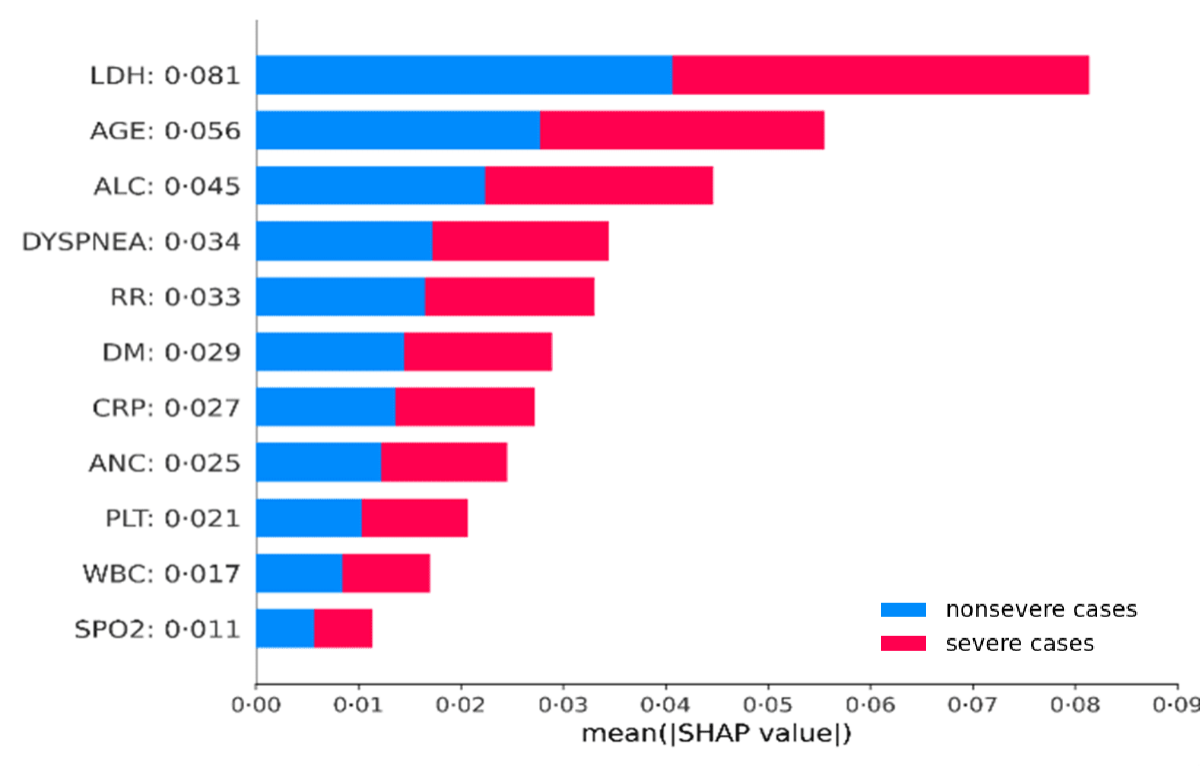
Average impact of 11 selected features in RIETS on COVID-19 severity prediction. To compute SHAP values based on the RIETS model, we properly modified KernelExplainer. Specific SHAP values are shown on the right of each feature name in the y-axis labels. ALC: absolute lymphocyte count; ANC: absolute neutrophil count; CRP: c-reactive protein; DM: diabetes mellitus; LDH: lactate dehydrogenase; PLT: platelet count; RIETS: Robust and Interpretable Early Triaging System; RR: respiratory rate; SHAP: Shapley additive explanations; SPO2: saturation of peripheral oxygen; WBC: white blood cell.

RIETS comprised 11 clinical and laboratory features: LDH, age, absolute lymphocyte counts (ALC), dyspnea, RR, DM, c-reactive protein (CRP), ANC, platelet counts (PLT), WBC, and SPO2. These features were ordered by their contribution to the severity prediction by using Shapley values (Figure 6). LDH was the highest ranked, followed by age, ALC, and dyspnea. In addition, pre-existing conditions (age, dyspnea, and DM) available at the time of admission were generally ranked higher relative to those of laboratory markers (CRP, ANC, PLT, WBC, and SPO2).

### Patient Clustering and Characterization

We identified 4 patient subgroups using DDRTree, a tree-based unsupervised learning technique, based on the features in RIETS: the upper-right group (URG), middle-right group (MRG), lower-right group (LRG), and lower-left group (LLG) (Figure 3A). Among the 4 subgroups, the URG comprised the largest proportion of patients at high risk for severity, followed by the MRG, LRG, and LLG. The majority of patients in the URG and MRG had dyspnea, were older than 60 years, and had elevated RR, CRP, and LDH (Figure 3B). High ANC and WBC were additionally observed in the MRG. Those in the LRG and LLG had elevated ANC, WBC, and PLT. There was a negligible variation in SPO2 or DM across the tree. Moreover, we compared the patient distribution per each variant period (original Alpha-dominant, Delta-dominant, and Omicron-dominant) and found no distinguishable pattern (Figure S5 in Multimedia Appendix 1).

## Discussion

### Principal Findings

We developed and validated RIETS, an ML-based prognostic model for severity among patients hospitalized with COVID-19, based on a temporally and geographically extensive cohort with heterogeneous feature distributions (Figure S6 in Multimedia Appendix 1). RIETS incorporates 11 promptly and routinely available features upon hospitalization and is intended to assist early patient triaging. RIETS provides risk estimates that indicate the odds for severity progression along with feature and patient interpretation. These outputs can support clinicians in making decisions for appropriate medical measures, such as the administration of antiviral medication, transportation to the intensive care unit, and proactive preparation of medical resources. Although several prognostic models with low risk of bias excel in analogous tasks [26-30], RIETS offers substantial improvements in several aspects owing to its discriminative power and novel interpretability (Table S2 and S5 in Multimedia Appendix 1).

According to PROBAST, RIETS can be regarded as a clinically applicable model with a low risk of bias because of its generalizability and methodologically rigorous procedure. First, RIETS can be generalized across diverse populations because it was developed and validated based on a large data set from a multicenter cohort (19 general and tertiary care hospitals) over the 3-year pandemic period (from January 2020 to August 2022). In contrast, previous prognostic models were either based on a large multicenter cohort during the early pandemic period [28,31,32] or a single center cohort covering a longer pandemic period [29,30,33]. Second, we executed a rigorous modeling procedure to establish RIETS. We exhaustively developed and simultaneously validated all possible combinations of candidate feature subsets and modeling algorithms (Figure S7 in Multimedia Appendix 1). Contrary to our study design, previous prognostic studies relied on a single feature selection approach (clinical consensus, least absolute shrinkage and selection operator regression, recursive feature elimination, and sequential forward selection) [26,28,32,34,35]. Since there is no one-size-fits-all solution in the model fine-tuning process [36], this comprehensive modeling procedure can provide engineering value in attaining optimal prediction with parsimonious feature usage.

RIETS demonstrated superior discriminative performance in contrast to previous prognostic models with a low risk of bias (RIETS: AUROC=0.937, 95% CI 0.935-0.938; previous studies: 95% CI 0.60-0.80) while maintaining comparable calibration (ICI=0.041 vs calibration-in-the-large=0.00; slope=0.96; Table S2 in Multimedia Appendix 1). It has high accuracies both in severe cases (sensitivity=0.869, 95% CI 0.864-0.875) and in nonsevere cases (specificity=0.867, 95% CI 0.865-0.869). This strength can offer considerable benefits in triaging situations because prompt treatment for critically ill patients is facilitated without the resource overutilization on less critical patients [37].

RIETS also can be broadly adaptable across health care systems. Unlike some well-established models based on advanced technology-based, expensive, and time-consuming features [30,32,38], RIETS comprises 11 readily available features obtainable from routine blood tests and patient-reported conditions at admission. Thus, it is interoperable even for health care systems in low- and middle-income countries and may offer significant operational benefits during resource allocation across the global population [12]. In addition, RIETS exhibited sustainable performance on Omicron cases, implicating its potential for transportability across new variant cases with differing virulency [39] and limited case availability (Figure S4 and Table S4 in Multimedia Appendix 1).

Lastly, RIETS offers substantial interpretability that may induce improvements in model reliability and operational workflow. To our knowledge, this is the first attempt in patient clustering and characterization amongst the COVID-19 prognostic models (Figures 3 and 4). Given that bias risks are inevitable in ML systems, the interpretability of RIETS can promote transparent feedback, mitigate those bias risks, and earn trust as a clinical decision support system [40,41]. Moreover, the patient clustering tool in RIETS provides clinicians with useful information for treatment planning and resource preparation. For instance, the graphical representations of patients can enable monitoring of the characteristics of incoming patients and facilitate the identification of representative clusters at the moment. This can be used to plan the customized patient care and to initiate the preemptive preparation of medical resources for those representative patient clusters.

### Limitations

This study has some limitations to be addressed. First, the study participants were patients hospitalized with COVID-19 in South Korea from January 2020 to August 2022. Hence, a further study with other ethnic and variant groups is recommended to validate the generalizability of RIETS. Second, the vaccination records were not accounted for during the analysis due to a high missing rate. Although vaccination often decreases the severity [42,43], a recent study showed that some vaccinated patients with certain chief complaints remained at high risk for severity [44]. This finding implicates that the impact of vaccination on severe case discrimination may not be large as long as the distributions of clinical signs remain similar across different variants. Lastly, the information on SARS-CoV-2 variants confirmed by viral whole genome sequencing were not available for each patient. We used variant dominant periods to define variant subcohorts while anticipating some misclassifications.

### Conclusions

We developed and validated RIETS, an ML-based COVID-19 severity prediction system, to promote the early triaging of hospitalized patients with COVID-19. RIETS demonstrated high prediction power and considerable reliability with low bias risk. Model development and validation on a nationwide, multicenter cohort implicated its generalizability. The use of routinely collected features for model construction facilitated its adaptability. Visual interpretations of model parameters and patients improved its usability and applicability. When incorporated into routine clinical practice, we anticipate RIETS to have a direct clinical impact for enabling efficient medical resource allocation as well as proactive patient care.

## Appendix

Multimedia Appendix 1 RIETS for COVID-19 Severity Prediction.

## Abbreviations

ALC: absolute lymphocyte counts
ANC: absolute neutrophil count
ASMD: absolute standardized mean difference
AUROC: area under the receiver operating characteristic curve
CRP: c-reactive protein
DCA: decision curve analysis
DDRTree: discriminative dimensionality reduction via learning a tree
DM: diabetes mellitus
DNN: deep neural network
FEM: feature engineering method
ICI: integrated calibration index
IRB: institutional review board
LDH: lactate dehydrogenase
LLG: lower-left group
LRG: lower-right group
ML: machine learning
MRG: middle-right group
PLT: platelet count
PROBAST: Diagnosis and Prediction Model Risk of Bias Assessment Tool
RF: random forest
RR: respiratory rate
SPO2: saturation of peripheral oxygen
TRIPOD: Transparent Reporting of a multivariable prediction model for Individual Prognosis
URG: upper-right group
WBC: white blood cell
WHO: World Health Organization
XGB: extreme gradient boosting

## Data Availability

This study used deidentified data from cloud-based data storage constructed by the Medical AI Center of Samsung Medical Center. Given the multisite origin of these data, access to data will require approval from the clinical sites and participating institutions; requests can be made to the corresponding author. We have deployed RIETS as a web-based open-source application to allow for vast usability from the wider public [45].
