# Supplementary material for: Development and Validation of a Robust and Interpretable Early Triaging Support System for Patients Hospitalized With COVID-19: Predictive Algorithm Modeling and Interpretation Study
Source: J Med Internet Res. 2024 Jan 11;26:e52134. doi: 10.2196/52134 (PMC10811577; doi:10.2196/52134)
Supplement: Multimedia Appendix 1 [file jmir_v26i1e52134_app1.docx]

**Appendix**

RIETS for COVID-19 Severity Prediction

| **Table of Contents** | **Page (s)** |
| --- | --- |
| **Abbreviation Box.** Comprehensive list of abbreviations and their definitions used throughout the study | 2 |
| **Supplementary Table S1.** Baseline characteristics in severe and non-severe cohorts | 3 |
| **Supplementary Table S2.** Comparison of the characteristics in RIETS to low bias risk and pre-existing prognostic models for severity | 4 |
| **Supplementary Table S3.** Comparison in Net Reclassification Improvements among prediction models | 5 |
| **Supplementary Table S4.** Performance of prediction models on omicron-variant cases in the external validation | 5 |
| **Supplementary Table S5.** A literature review on past models developed to predict COVID-19 severity and mortality | 6-7 |
| **Supplementary Figure S1.** Six feature engineering methods based on two ML algorithms and four feature importance measures to generate candidate features | 8 |
| **Supplementary Figure S2.** Reclassification Plots | 9 |
| **Supplementary Figure S3.** Discriminative performance for Omicron-variant cases in the external validation | 10 |
| **Supplementary Figure S4.** Empirical distributions of AUROC scores for ML algorithms across different class weights | 11 |
| **Supplementary Figure S5.** Characterization of patient subgroups for each variant | 12 |
| **Supplementary Figure S6.** Distribution of each feature in RIETS across centers | 13-14 |
| **Supplementary Figure S7.** Empirical distributions of AUROC scores in internal and external validations | 15 |
| **Supplementary Methods S1.** Study setting and design | 16 |
| **Supplementary Methods S2.** Model Development and Validation | 16-17 |
| **Supplementary Methods S3.** Model Performance Visualization and Interpretation | 17 |
| **Supplementary Methods S4.** Model Transportability to New Variant Dominant Period | 17-18 |
| **Supplementary Methods S5.** Statistical analysis | 18 |
| **Supplementary File S1.** TRIPOD Checklist for Prediction Model Development and Validation | 19 |
| Supplementary File S2. PROBAST Guideline for Prediction model study Risk Of Bias Assessment Tool | 20-27 |
| Supplementary References | 28 |

**Abbreviation Box:** Comprehensive list of abbreviations and their definitions used throughout the study

| **Study Design and Reporting** | |
| --- | --- |
| DC | development cohort |
| VC | validation cohort |
| RIETS | Robust and Interpretable Early Triaging System |
| **Machine Learning and Statistical Methods** | |
| ASMD | absolute standardized mean difference |
| CDSS | clinical decision support system |
| CV | cross-validation |
| ML | machine learning |
| **Feature Interpretation Methods** | |
| DDRTree | discriminative dimensionality reduction via learning a tree |
| SHAP | shapley additive exPlanations |
| **Feature Engineering Methods** | |
| FEMs | feature engineering methods |
| RF-MDIFI | random forest-based mean decreases in Gini impurity feature importance |
| RF-PFI | random forest-based permutation feature importance |
| RF-Shapley | random forest-based Shapley values |
| XGB-BFI | eXtreme Gradient Boosting-based built-in feature importance |
| XGB-PFI | eXtreme Gradient Boosting-based permutation feature importance |
| XGB-Shapley | eXtreme Gradient Boosting-based Shapley values |
| **Machine Learning Algorithms** | |
| DNN | deep neural network |
| GBM | gradient boosting machine |
| MLR | multivariable logistic regression |
| RF | random forest |
| SVM | support vector machine |
| XGB | eXtreme gradient boosting |
| **Clinical and Laboratory Biomarkers** | |
| ALC | absolute lymphocyte counts |
| ANC | absolute neutrophil count |
| CRP | c-reactive protein |
| DM | diabetes mellitus |
| LDH | lactate dehydrogenase |
| PLT | platelet counts |
| RR | respiratory rate |
| SPO2 | saturation of peripheral oxygen |
| WBC | white blood cell counts |
| **Model Performance Metrics** | |
| AUROC | area under receiver operating characteristic curve |
| CI | confidence intervals |
| DCA | decision curve analysis |
| DOR | diagnostic odds ratio |
| ICI | integrated calibration index |
| LRP | likelihood ratio positive |
| LRN | likelihood ratio negative |
| NPV | negative predicted values |
| PPV | positive predicted values |

**Supplementary Table S1: Baseline characteristics in severe and non-severe cohorts**

|  | **Severe Patients** | **Non-severe Patients** | **ASMD** |
| --- | --- | --- | --- |
|  | **(N: 839)** | **(N: 5,106)** |  |
| **Patient Characteristics** | | | |
| Age (years) | 70 (60-75) | 55 (40-70) | 0·733 |
| Male Sex (%) | 499 (59·5%) | 2,388 (46·8%) | 0·255 |
| Diagnosed Date | -2 (-5-0) | 0 (-1-0) | 0·591 |
| **Comorbidities (%)** | | | |
| Hypertension | 462 (55·1%) | 1,652 (32·4%) | 0·462 |
| Diabetes Mellitus | 345 (41·1%) | 904 (17·7%) | 0·524 |
| CVD | 106 (12·6%) | 402 (7·9%) | 0·158 |
| Cancer | 75 (8·9%) | 402 (7·9%) | 0·038 |
| Others | 401 (47·8%) | 1,841 (36·1%) | 0·239 |
| **Clinical Symptoms (%)** | | | |
| Fever | 364 (43·4%) | 2,002 (39·2%) | 0·085 |
| Cough | 317 (37·8%) | 2,306 (45·2%) | 0·150 |
| Sputum | 181 (21·6%) | 1,321 (25·9%) | 0·101 |
| Dyspnea | 487 (58·0%) | 831 (16·3%) | 0·902 |
| Myalgia | 92 (11·0%) | 1,296 (25·4%) | 0·381 |
| Sorethroat | 59 (7·0%) | 1,083 (21·2%) | 0·420 |
| Loss of Sensor | 31 (3·7%) | 299 (5·9%) | 0·102 |
| GI symptom | 53 (6·3%) | 419 (8·2%) | 0·073 |
| **Vital Sign** | | | |
| BT (℃) | 36·7 (36·4-37·4) | 36·6 (36·3-37·2) | 0·187 |
| SBP (mmHg) | 130 (116-145) | 129 (116-140) | 0·056 |
| DBP (mmHg) | 73 (65-83) | 80 (70-88) | 0·447 |
| PR (%) | 85 (73-98) | 84 (75-95) | 0·116 |
| RR (%) | 22 (20-25) | 20 (18-20) | 0·911 |
| SPO2 (%) | 96 (93-98) | 97 (96-98) | 0·705 |
| **Blood biochemistry** | | | |
| WBC (10^3^/µL) | 7·2 (5·0-10·2) | 5·1 (4·0-6·5) | 0·693 |
| ANC (10^3^/µL) | 5·9 (3·8-8·8) | 3·3 (2·3-4·8) | 0·971 |
| ALC (10^3^/µL) | 0·8 (0·5-1·1) | 1·3 (0·9-1·7) | 0·497 |
| PLT (10^3^/µL) | 178 (131-237) | 203 (162-249) | 0·295 |
| CRP (mg/dL) | 9·1 (3·7-15·0) | 0·8 (0·2-3·5) | 0·248 |
| LDH (U/L) | 496 (364-723) | 295 (213-407) | 0·726 |
| **Hospital Location and Type** | | | |
| Metropolitan area general | 56 (6·7%) | 691 (13·5%) | 0.231 |
| Metropolitan area tertiary care | 421 (50·2%) | 964 (18·9%) | 0.675 |
| Non-metropolitan area general | 48 (5·7%) | 212 (4·2%) | 0.073 |
| Non-metropolitan area tertiary care | 314 (37·4%) | 3,239 (63·4%) | 0.526 |

Data are median (IQR) or n (%). ASMD=absolute standardized mean difference. CVD=cardio-vascular disease. GI= gastrointestinal. BT=body temperature. SBP=systolic blood pressure. DBP=diastolic blood pressure. PR=pulse rate. RR=respiratory rate. SPO2=saturation of peripheral oxygen. WBC= white blood cell. ANC=absolute neutrophil count. ALC=absolute lymphocyte count. PLT= platelet count. CRP=c-reactive protein. LDH=lactate dehydrogenase

**Supplementary Table S2. Comparison of the characteristics in RIETS to low bias risk and pre-existing prognostic models for severity**

| **Author** | **Model Name** | **Predictors** | **EV Performance (AUROC)** | **Calibration** | **Interpretability** | **Study Setting (# of sites)** | **Sample size** | **Collection Period** | **Feature Selection Technique** | **Modeling Process** | **Validation Method** |
| --- | --- | --- | --- | --- | --- | --- | --- | --- | --- | --- | --- |
| Baek et al. | **RIETS** | LDH, age, ALC, dyspnea, RR, DM, CRP, ANC, PLT, WBC, SPO2 | **0.937 [0.935-0.938]** | **ICI: 0.041** | Patient clustering and characterization; feature interpretation | South Korea (19) | D: 4,365 (3,760 NS vs. 605 S) EV: 1,580 (1,346 NS vs. 234 S) | D: Jan 26 2020 to Aug 29 2022 EV: Jan 5 2020 to Aug 23 2022 | Identification of stably-selected candidate feature subsets by applying six ML-based feature selection techniques | Exhaustive search based on 60 feature subsets and six ML-based algorithms (DNN, MLR, RF, XGB, GBM, SVM) | Internal Validated with five iterations of 5-fold cross validation  External validated with bootstrapped cohort from three sites |
| Royal College of Physicians | NEWS2* | RR, SPO2, SBP, PR, consciousness, BT | 0.78 [0.73-0.83] | NA | NA | UK (1) | EV: 411 (231 NS vs. 180 S) | EV: Feb 1 2020 to April 30 2020 | Clinical consensus | MLR | NA |
| Subbe et al. | MEWS* | SBP, PR, RR, BT, AVPU score | 0.60 [0.54-0.65] | NA | NA | UK (1) | EV: 411 (231 NS vs. 180 S) | EV: Feb 1 2020 to April 30 2020 | Clinical consensus | MLR | NA |
| Gupta et al. | ISARIC4C Deterioration | age, sex, nosocomial infection, GCS score, SPO2, room air or oxygen therapy, RR, urea, CRP, ALC, radiographic chest infiltrates | 0.77 [0.76-0.78] | CITL: 0.00 [-0.05-0.05] Slope: 0.96 [0.91-1.01] | NA | UK (260) | D: 66,705 (38,565 NS vs. 28,140 S) EV: 8,239 (4,455 NS vs. 3,784 S) | D: Feb 6 2020 to Aug 2026 2020 EV: Feb 6 2020 to Aug 2026 2020 | Backward elimination of the priori candidate variables | MLR | Internally validated with regional-based masking cross validation process Externally validated with cohort from a single site |
| Carr et al. | CARR model | NEWS2 score, supplemental oxygen flow rate, SPO2, urea, age, CRP, estimated GFR, ANC, NLR | 0.735 [0.715-0.757] | Scores not shown; Poorly calibrated | NA | International (5 UK, 1 Norway, 2 China) | D: 1,276 (887 NS vs. 389 S) EV: 6,237 (4,929 NS vs. 1,308 S) | D: March 1 2020 to April 31 2020 EV: Feb 1 2020 to August 26 2020 | Automatic variable selection by LASSO Three candidate feature subsets (NEWS2 only, NEWS2+age, NEWS2+eight features) | LASSO | Internally validated with nested cross-validation (10-folds repeated 1000 times) Externally validated with cohort from seven sites |
| Goodacre et al. | PRIEST | Age, sex, RR, SBP, SPO2, performance status, consciousness, history of renal impairment, respiratory distress | 0.80 [0.79-0.81] | Scores not shown; Poorly calibrated | NA | UK (53) | D: 11,773 (9,063 NS vs. 2,710 S) EV: 9,118 (6,638 NS vs. 2,480 S) | D: Mar 26 2020 to May 28 2020 EV: Mar 26 2020 to May 28 2020 | LASSO and Clinical consensus | MLR | Random split (8:2 ratio) of derivation and validation cohorts, then validated with LASSO |

*Pre-existing models prevalently used for patient risk stratification; the reported model performance was externally evaluated by Gupta et al. RIETS=Robust and Interpretable Early Triaging System. NEWS2=National Early Warning Score. MEWS=modified early warning score. ISARIC4C=International Severe Acute Respiratory and Emerging Infections Consortium Coronavirus Clinical Characterization Consortium. PRIEST=Pandemic Respiratory Infection Emergency System Triage. EV=external validation. D=development. NS=non-severe. S=severe. ICI=integrated calibration index. CITL=calibration-in-the-large. UK=United Kingdom. LDH=lactate dehydrogenase. ALC=absolute lymphocyte count. RR=respiratory rate. DM=diabetes mellitus. CRP=c-reactive protein. ANC=absolute neutrophil count. PLT=platelet count. WBC=white blood cell count. SPO2=saturation of peripheral oxygen. SBP=systolic blood pressure. PR=pulse rate. BT=body temperature. AVPU=alert verbal pain unresponsive. GCS=Glasgow coma scale. GFR=glomerular filtration rate. NLR=neutrophil lymphocyte ratio.

**Supplementary Table S3. Comparison in Net Reclassification Improvements among prediction models**

|  | **NRI** | **NRI+ (Severe)** | **NRI- (Non-Severe)** |
| --- | --- | --- | --- |
| **RIETS vs MLR** | 0·0053 | -0·0458 | 0·0511 |
| **RIETS vs RF** | 0·0137 | -0·0153 | 0·0290 |
| **RIETS vs XGB** | 0·0614 | 0·0000 | 0·0614 |
| **RIETS vs GBM** | 0·0394 | 0·0076 | 0·0317 |
| **RIETS vs SVM** | 0·0290 | 0·0076 | 0·0214 |

RIETS=Robust and Interpretable Early Triaging System. NRI=net reclassification improvements. MLR=multivariable logistic regression. RF=random forest. XGB=eXtreme gradient boosting. GBM=gradient boosting machine. SVM=support vector machine.

**Supplementary Table S4. Performance of prediction models on omicron-variant cases in the external validation**

|  | **Predictive Measures** | | | | | | | |  |
| --- | --- | --- | --- | --- | --- | --- | --- | --- | --- |
| **Model** | **AUROC** | **Sensitivity** | **Specificity** | **PPV** | **NPV** | **LRP** | **LRN** | **DOR** | **Cut-Off** |
| RIETS-Ensemble | **0·903 [0·897-0·910]** | 0·897 [0·881-0·913] | 0·831 [0·814-0·849] | 54·56 [52·28-56·84] | 97·65 [97·29-98·01] | 6·70 [6·08-7·33] | 0·12 [0·10-0·14] | **56·22 [49·83-62·61]** | 0·197 |
| RIETS-All | 0·834 [0·825-0·844] | 0·876 [0·856-0·896] | 0·720 [0·701-0·740] | 41·22 [39·06-43·38] | 96·76 [96·30-97·22] | 4·07 [3·34-4·80] | 0·16 [0·14-0·19] | 29·00 [25·89-32·11] | 0·036 |
| RIETS-Omicron | 0·813 [0·800-0·826] | 0·912 [0·895-0·928] | 0·697 [0·683-0·712] | 39·14 [37·44-40·85] | 97·67 [97·30-98·04] | 3·37 [3·02-3·73] | 0·12 [0·10-0·14] | 33·79 [30·40-37·19] | 0·391 |

All results, 95% CIs in parentheses, were computed from bootstrapping external validation cohorts 100 times with replacements. Youden’s Index was used to determine optimal cut-off point. RIETS=Robust and Interpretable Early Triaging System. AUROC=area under receiver operating characteristic curve. PPV=positive predictive value. NPV=negative predictive value. LRP=likelihood ratio positive. LRN=likelihood ratio negative. DOR=diagnostic odds ratio.

**Supplementary Table S5. A literature review on past models developed to predict COVID-19 severity and mortality**

| **Date** | **Journal** | **Author** | **Model Name** | **Outcomes** | **Predictors** | **Performance (AUROC)** | **Study Setting (# of sites)** | **Center Type** | **Patient Cohort Size** | **Collection Period** | **Feature Selection Technique** | **Modeling Process** | **Data Imputation** |
| --- | --- | --- | --- | --- | --- | --- | --- | --- | --- | --- | --- | --- | --- |
| N/A | Not yet published | Baek S W, et al. | RIETS | Severity | LDH, age, ALC, dyspnea, RR, DM, CRP, ANC, PLT, WBC, SPO2 | Internal: 0.891 (0.889-0.892) External: 0.937 (0.935-0.938) | South Korea (19) | Tertiary care and general hospitals | D: 4,365 (3,760 NS vs. 605 S) EV: 1,580 (1,346 NS vs. 234 S) | D: Jan 26 2020 to Aug 29 2022 EV: Jan 5 2020 to Aug 23 2022 | Identified stably-selected feature from 6 feature engineering methods | Exhaustive search based on 60 feature subsets and six algorithms (DNN, MLR, RF, XGB, GBM, SVM) | Complete-case analysis |
| 2001 Oct | QJM | Subbe et al. | MEWS* | Severity | SBP, PR, RR, BT, AVPU score | External: 0.60 (0.54-0.65) | UK (1) | General hospital | EV: 411 (231 NS vs. 180 S) | EV: Feb 1 2020 to April 30 2020 | Not performed | MLR | MICE |
| 2017 Dec | Book | Royal College of Physicians | NEWS2* | Severity | RR, SPO2, SBP, PR, Consciousness, BT | External: 0.78 (0.73-0.83) | UK (1) | General hospital | EV: 411 (231 NS vs. 180 S) | EV: Feb 1 2020 to April 30 2020 | Not performed | MLR | MICE |
| 2020 June | ERJ | Wu G, et al. | Wu Model | Severity | age, ALC, CRP, LDH, creatinine kinase, urea, and calcium | Internal: 0.86 (0.82-0.90)  External: 0.90 (0.82-0.98) | China, Italy, Belgium (8) | General hospitals | 299 (228 NS vs. 71 S) | Dec 23 2019 to Mar 21 2020 | Recursive feature elimination | MLR | ADASYN |
| 2021  Jan | Lancet Respiratory Medicine | Gupta et al. | ISARIC4C Deterioration | Severity | age, sex, nosocomial infection, GCS score, SPO2, breathing room air or oxygen therapy, RR, urea, CRP, ALC, presence of radiographic chest infiltrates | External: 0.77 (0.76-0.78) | UK (260) | General hospitals | D: 66,705 (38,565 NS vs. 28,140 S) EV: 8,239 (4,455 NS vs. 3,784 S) | D: Feb 6 2020 to Aug 2026 2020 EV: Feb 6 2020 to Aug 2026 2020 | Backward elimination of the priori candidate variables | MLR | MICE |
| 2021  Jan | BMC Medicine | Carr et al. | CARR model | Severity | NEWS2 score, supplemental oxygen flow rate, SPO2, urea, age, CRP, estimated glomerular filtration rate, ANC, NLR | External: 0.735 (0.715-0.757) | UK, Norway, China (8) | General hospitals | D: 1,276 (887 NS vs. 389 S) EV: 6,237 (4,929 NS vs. 1,308 S) | D: March 1 2020 to April 31 2020 EV: Feb 1 2020 to August 26 2020 | LASSO estimator (automatic variable selection) | LASSO | KNN Imputation |
| 2021  Jan | PLoS One | Goodacre et al. | PRIEST | Severity | Age, sex, RR, SBP, SpO2, Performance status, consciousness, history of renal impairment, respiratory distress | External: 0.80 (0.79-0.81) | United Kingdom (53) | General hospitals | D: 11,773 (9,063 NS vs. 2,710 S) EV: 9,118 (6,638 NS vs. 2,480 S)) | D: Mar 26 2020 to May 28 2020 EV: Mar 26 2020 to May 28 2020 | LASSO and Clinical Consensus | MLR | MICE |
| 2021 April | NPJ Digital Medicine | Zhou J, et al. | Zhou Model | Severity | age, gender, comorbidities, medication records, and laboratory examination results | Internal: 0.86 (0.82-0.91) External: 0.89 (0.85-0.93) | D: China (42) EV: China (1) | D: local public database  EV: general Hospital | D: 4,442 (4,233 NS vs. 209 S) EV: 202 | D: Jan 1 2020 to Aug 22 2020 EV: Feb 10 2020 to March 10 2020 | Univariate logistic regression analysis | MLR | Not performed |
| 2021 November | Scientific Reports | An C, et al. | An Model | Severity | age, sex, symptoms, underlying disease, blood test results, and vital signs | Internal: 0.907 (0.884-0.929) External: 0.871 (0.834-0.910) | South Korea (2) | D: public dataset EV: hospital | 6,041 (5,596 development vs. 445 validation) | D: until April 30 2020 EV: Dec 19 2020 to March 16 2021 | LASSO and RF variable importance | LASSO | Not performed |
| 2022 March | Int J Environ Res Public Health | Alrajhi A, et al. | Alrajhi Model | Severity | SPO2, RR, Age, BT, BMI, heart rate, BMI, weight, height, blood type, clinical symptoms, comorbidities | Internal: 0.87 | Saudi Arabia (1) | Tertiary care hospital | D: 1,848 (1,715 NS vs. 133 S) EV: 185 (172 NS vs. 13 S) | D: March 2020 to April 6 2021 EV: April 2021 to May 2021 | Not performed | MLR, RF, XGB, Extra Trees | KNN imputation |
| 2022 March | PLOS Digit Health | Bhatia S, et al. | Bhatia Model | Severity | age, urea, hs-CRP, DD, indirect bilirubin, AST, monocytes, RBC, WBC, ferritin | External: 0.93 | India (2) | General hospitals | D: 264 (118 NS vs. 146 S) EV: 67 (30 NS vs. 37 S) | July 13 2020 to December 2020 | Top 10 variables from SHAP plot | XGB | Random undersampling for the majority class |
| 2022 November | Diagnostics | Lodato I, et al. | Lodato Model | Severity | age, CRP, LDH, hs-Tnl, ALC, creatinine, urea, ANC | Internal: 91% (Accuracy) | China (1) | General hospital | 418 (321 NS, 44 mild, 15 serious, 38 critical) | Feb 12, 2020 to Aug 25 2020 | Mutual Information Classification | Decision tree, RF, GBM, RUSBoost | SMOTE |
| 2023 April | ERJ Open Res | Nguyen H T T, et al. | Nguyen Model | Severity | ALT, albumin, AST, creatinine, IL-6, DD, CRP, ferritin, PLT, SPO2, urea, WBC | Internal: 92% (Accuracy) External:86% (Accuracy) | Vietnam, Denmark (2) | General hospitals | D: 261 (61 NS vs. 200 S) EV: 132 (34 NS vs. 98 S) | D: July 26 2021 to Nov 22 2021 EV: Mar 25 2020 to Feb 2 2021 | Random forest analysis and clinical consensus | RF | Not described |
| 2016 Feb | JAMA | Seymour et al. | qSOFA* | Mortality | systolic hypotension, tachypnoea, altered mentation | External: 0.60 (0.55-0.65) | UK (1) | General hospital | EV: 411 (231 NS vs. 180 S) | EV: Feb 1 2020 to April 30 2020 | Not performed | MLR | MICE |
| 2020 September | BMJ | Knight S, et al. | 4C Mortality | Mortality | age, sex, number of comorbidities, RR, SPO2, level of consciousness, urea, CRP | Internal: 0.79 (0.78-0.79) External: 0.77 (0.76-0.77) | UK (260) | General hospitals | D: 35,463 (24,037 survivors vs. 11,426 non-survivors) EV: 22,361 (15,632 survivors vs. 6,729 non-survivors) | D: Feb 6 2020 to May 20 2020 EV: May 21 2020 to Jun 29 2020 | Criterion based approach to variable selection | MLR | MICE |
| 2020 October | Nature Communications | Gao Y, et al. | MRPMC | Mortality | DD, albumin, SPO2, BUN, RR, ALC, PLT, Age, Sex, Fever, Number of comorbidities, Sputum, CKD, Consciousness | Internal: 0.961 (0.946-0.978) EV: 0.976 (0.961-0.991); 0.925 (0.876-0.973) | China (3) | General hospitals | D: 1243 (1068 survivors vs. 175 non-survivors) EV: 917 (838 survivors vs. 79 non-survivors) | D: Jan 27 2020 to Mar 21 2020 EV: Jan 30 2020 to Mar 21 2020 | LASSO regression | MLR, SVM, GBM, DNN | MICE |
| 2020 December | Scientific Reports | Gue Y X, et al. | Gue Model | Mortality | age, sex, PLT, international normalized ratio, GCS score, RR, and BP | Internal: 0.793 (0.745-0.841) | UK (1) | General hospital | 316 (171 survivors vs. 145 non-survivors) | Mar 10 2020 to May 30 2020 | Not performed | MLR | Complete-case analysis |
| 2021 February | Scientific Reports | Jimenez-Solem E, et al. | Jimenez Model | Mortality | age, gender, BMI, comorbidities | Internal: 0.902 External: 0.742 | Denmark (2) | Public dataset | 3,944 (3620 survivors vs. 324 non-survivors) | Mar 1 2020 to Jun 16 2020 | Not performed | SVM, MLR, GBM, DNN, RF | KNN imputation |
| 2022 October | Scientific Reports | Cisterna-Garcia A, et al. | Cisterna Model | Mortality | age, sex, number of comorbidities, osteoarthritis, obesity, depression, and renal failure | Internal: 0.94 | Spain (1) | Public dataset | 81,386 (81,386 outpatient, 4,736 non-ICU inpatient, 745 ICU inpatient) | Jan 4 2020 to Feb 4 2021 | Not performed | MLR, RF | LR-IPIP |
| 2023 Mar | BMC Med Inform Decis Mak | Raman G, et al. | Raman Model | Mortality | SPO2, pulse oximetry, O2 flow rate, ferritin, CRP, BUN, RR, AST, albumin, PLT, ANC, hs-Tnl, WBC, age, DD, MCV, creatinine, BMI, ALT | Internal: 0.82 | USA (1) | Tertiary care hospital | 1,795 (1,639 non-survivors vs. 156 survivors) | May 2020 to March 2022 | Random forest-based permutation feature importance | RF | Not described |

D=development. EV=external validation. NS=non-severe. S=severe. MLR=multivariable logistics regression. RF=random forest. SVM=support vector machine. GBM=gradient boosting machine. DNN=deep neural network. XGB=eXtreme gradient boosting. LASSO=least absolute shrinkage and selection operator. AUROC=area under the receiver operating characteristic curve. LDH=lactate dehydrogenase. ALC=absolute lymphocyte count. RR=respiratory rate. DM=diabetes mellitus. CRP=c-reactive protein. ANC=absolute neutrophil count. PLT=platelet count. WBC=white blood cell count. SPO2=saturation of peripheral oxygen. SBP=systolic blood pressure. PR=pulse rate. BT=body temperature. AVPU=alert verbal pain unresponsive. GCS=Glasgow coma scale. GFR=glomerular filtration rate. NLR=neutrophil lymphocyte ratio. MICE=multiple imputation with chained equations. ALT=alanine aminotransferase. AST=aspartate aminotransferase. Hs-CRP=high-sensitivity c-reactive protein. Hs-tnl=high-sensitivity troponin I. IL-6=interleukin-6. DD=d-dimer. LR-IPIP=Logistic regression based identical partitions for imbalance problems. ADASYN=adaptive synthetic sampling approach for imbalanced learning. SMOTE=Synthetic Minority Oversampling Technique.

**Supplementary Figure S1. Six feature engineering methods based on two ML algorithms and four feature importance measures to generate candidate features**


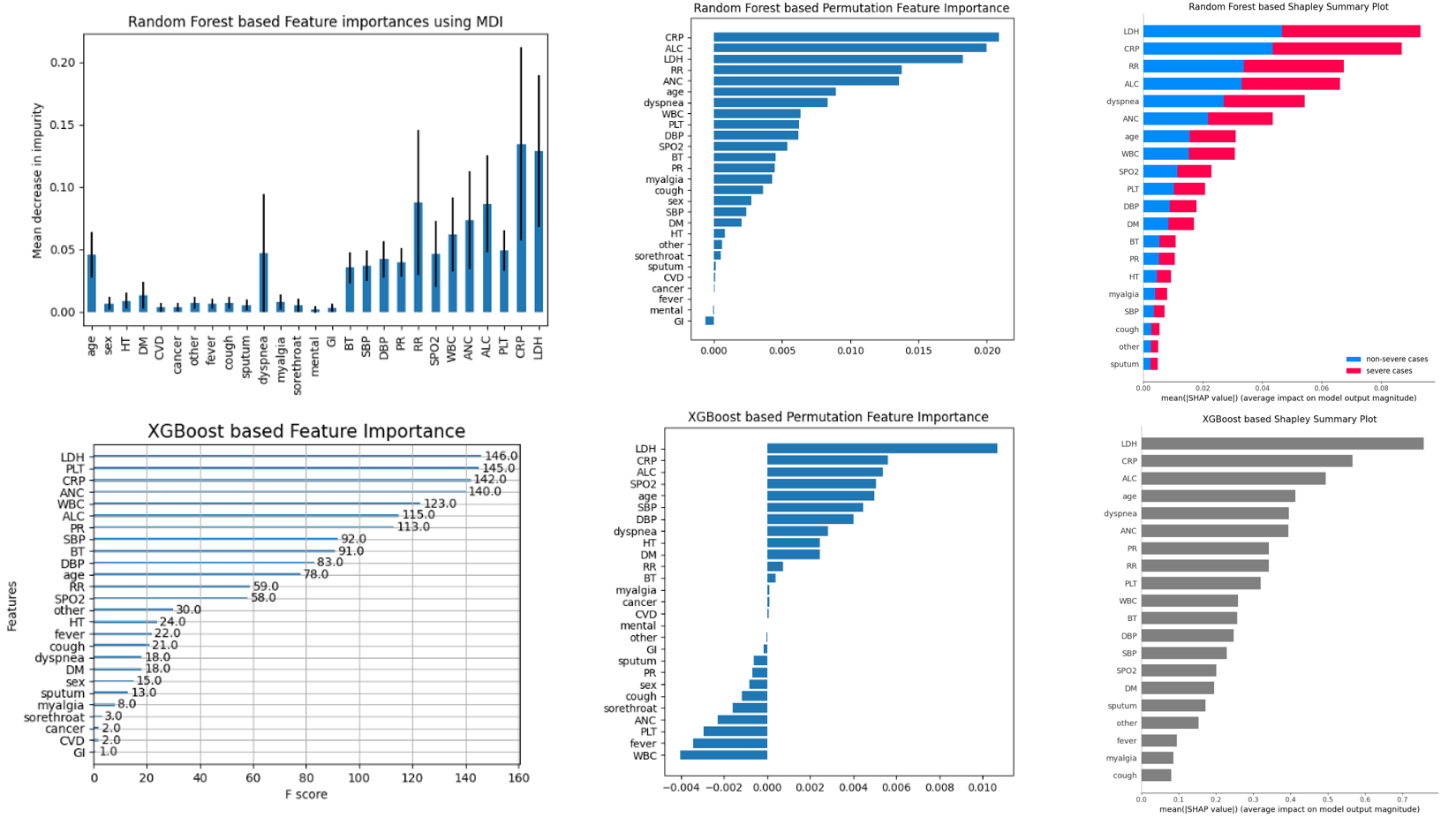
MDI=mean decrease in impurity. CVD=cardio-vascular disease. GI= gastrointestinal. BT=body temperature. SBP=systolic blood pressure. DBP=diastolic blood pressure. PR=pulse rate. RR=respiratory rate. SpO2=saturation of peripheral oxygen. WBC= white blood cell. ANC=absolute neutrophil count. ALC=absolute lymphocyte count. PLT= platelet count. CRP=c-reactive protein. LDH=lactate dehydrogenase.

**Supplementary Figure S2. Reclassification Plots**

**(A) comparing the predictive performances between RIETS and MLR models. (B) comparing the predictive performances between RIETS and RF models.**

**A B**

**
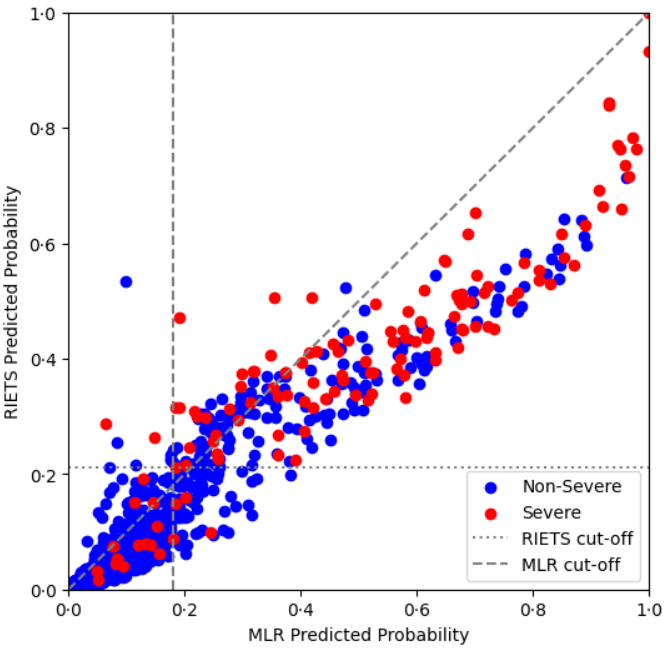
** **
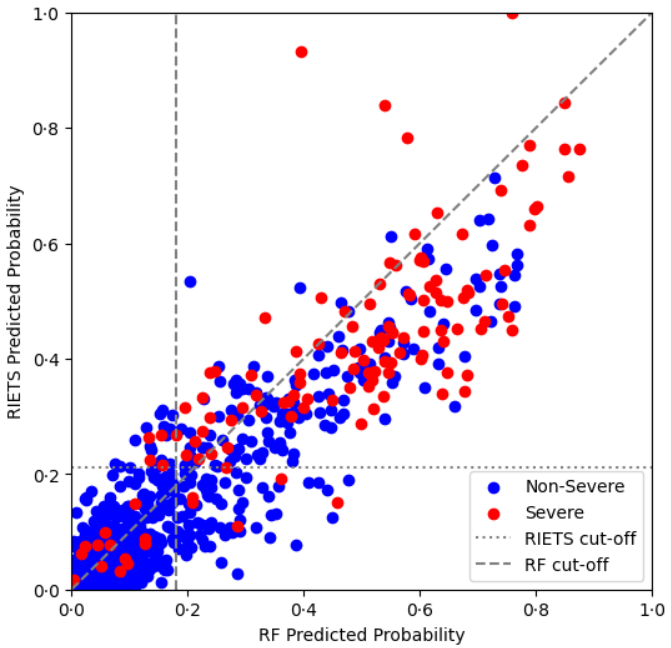
**

*Horizontal and vertical dotted lines represent Youden’s index-based cut-off point for each respective algorithm. RIETS=Robust and Interpretable Early Triaging System. MLR=multivariable logistic regression. RF=random forests.

**Supplementary Figure S3. Empirical distributions of AUROC scores for ML algorithms across different class weights**

**(A) internal validation results. (B) external validation results.** Empirical distributions of area under receiver operating characteristic curve (AUROC) scores were estimated for three ML algorithms across class weights from 1 to 10 all 60 feature subsets during the internal (IV) and external validations (EV). MLR=multivariable logistic regression. DNN=deep neural network. RF=random forest

**A**

**
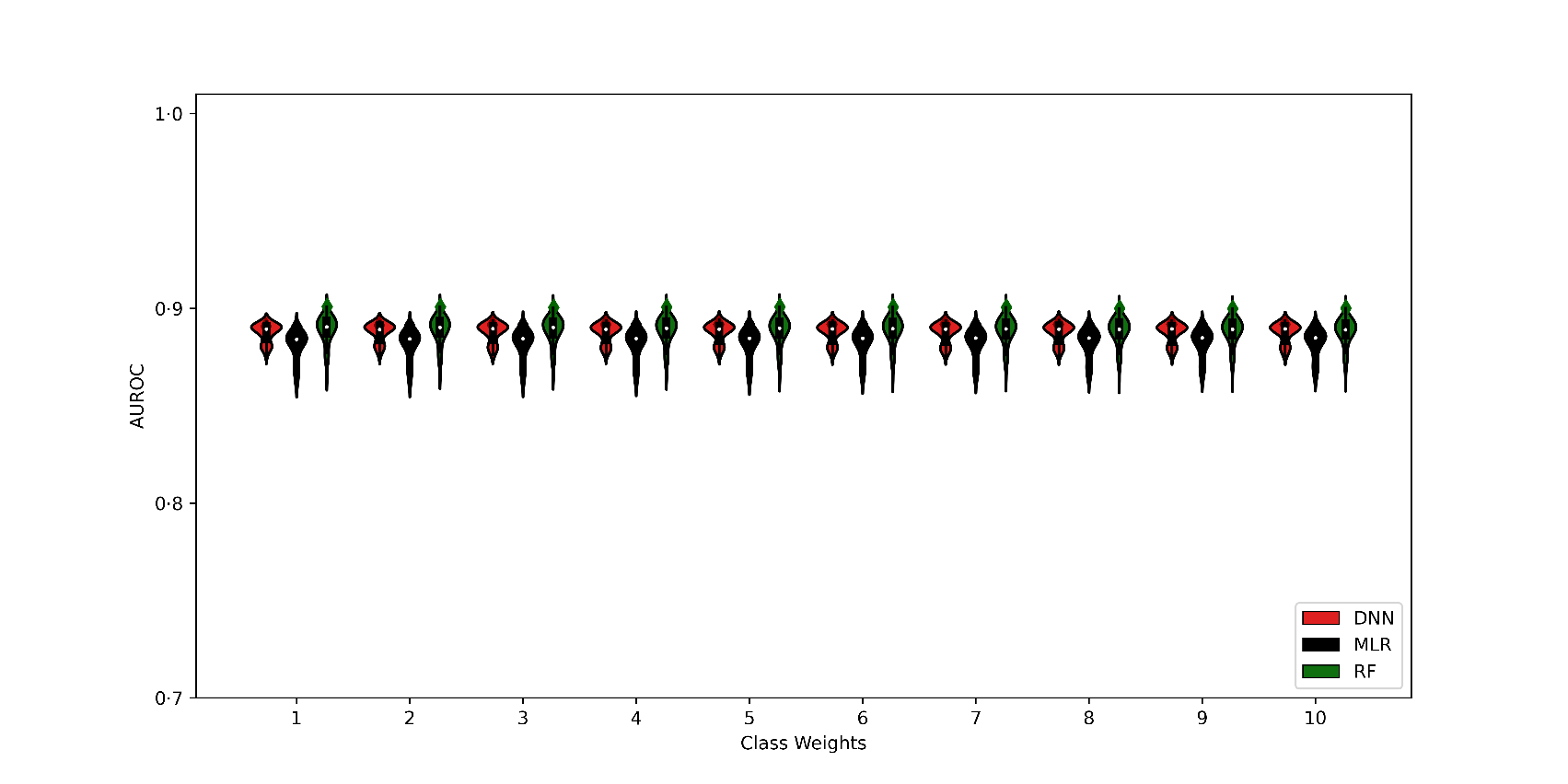
**

**B**

**
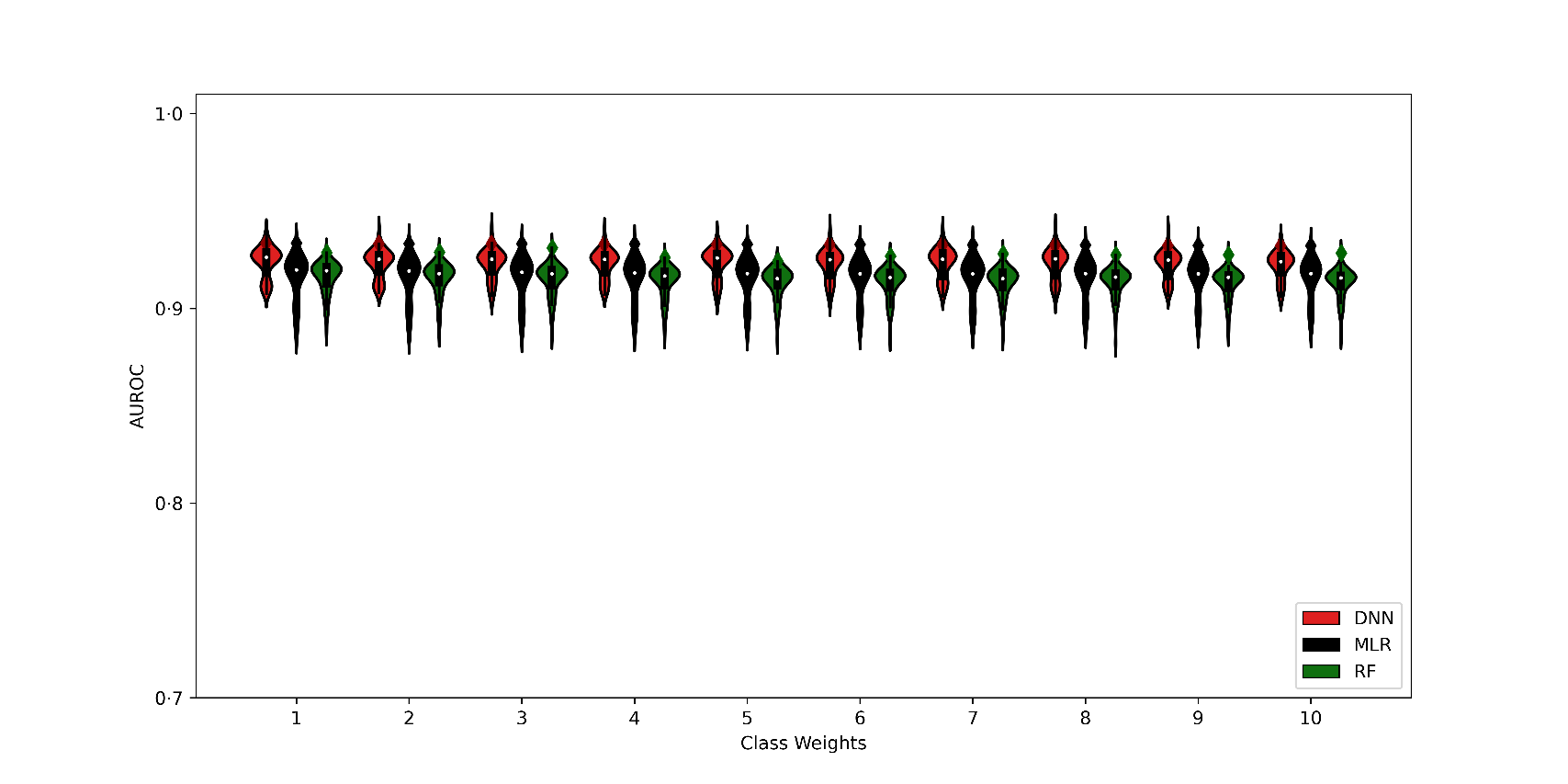
**

**Supplementary Figure S4. Discriminative performance for Omicron-variant cases in the external validation**


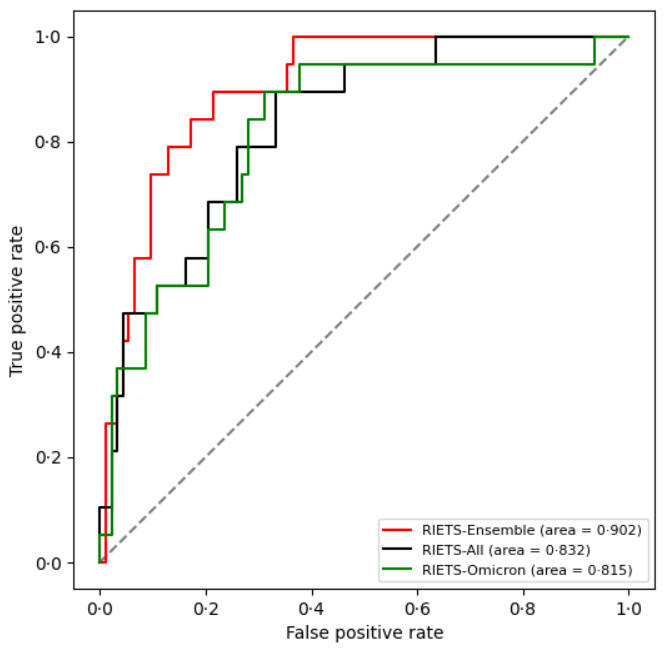


Used DNN-based final prediction model, RIETS, to develop each model. RIETS-Ensemble model was developed by combining the prediction probabilities of the three models based on Alpha&Omicron, Delta&Omicron, and Omicron development cohorts; RIETS-All model was developed by using whole development cohort; RIETS-Omicron model was developed by using only Omicron development cohort. Patient size (N): whole development cohort, 4,365 (Alpha development cohort, 2,622; Delta development cohort, 1,406; Omicron development cohort, 337); whole validation cohort, 1580 (Alpha validation cohort, 840; Delta validation cohort, 628; Omicron validation cohort, 112). RIETS=Robust and Interpretable Early Triaging System. DNN=deep neural network.

**Supplementary Figure S5. Characterization of patient subgroups for each variant**

*Dark red color indicates high concentration and light green color indicates low concentration of each corresponding feature.

**
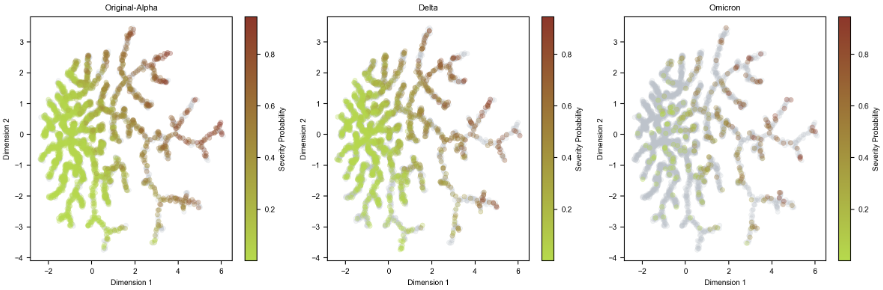
**

**Supplementary Figure S6. Distribution of each feature in RIETS across centers**


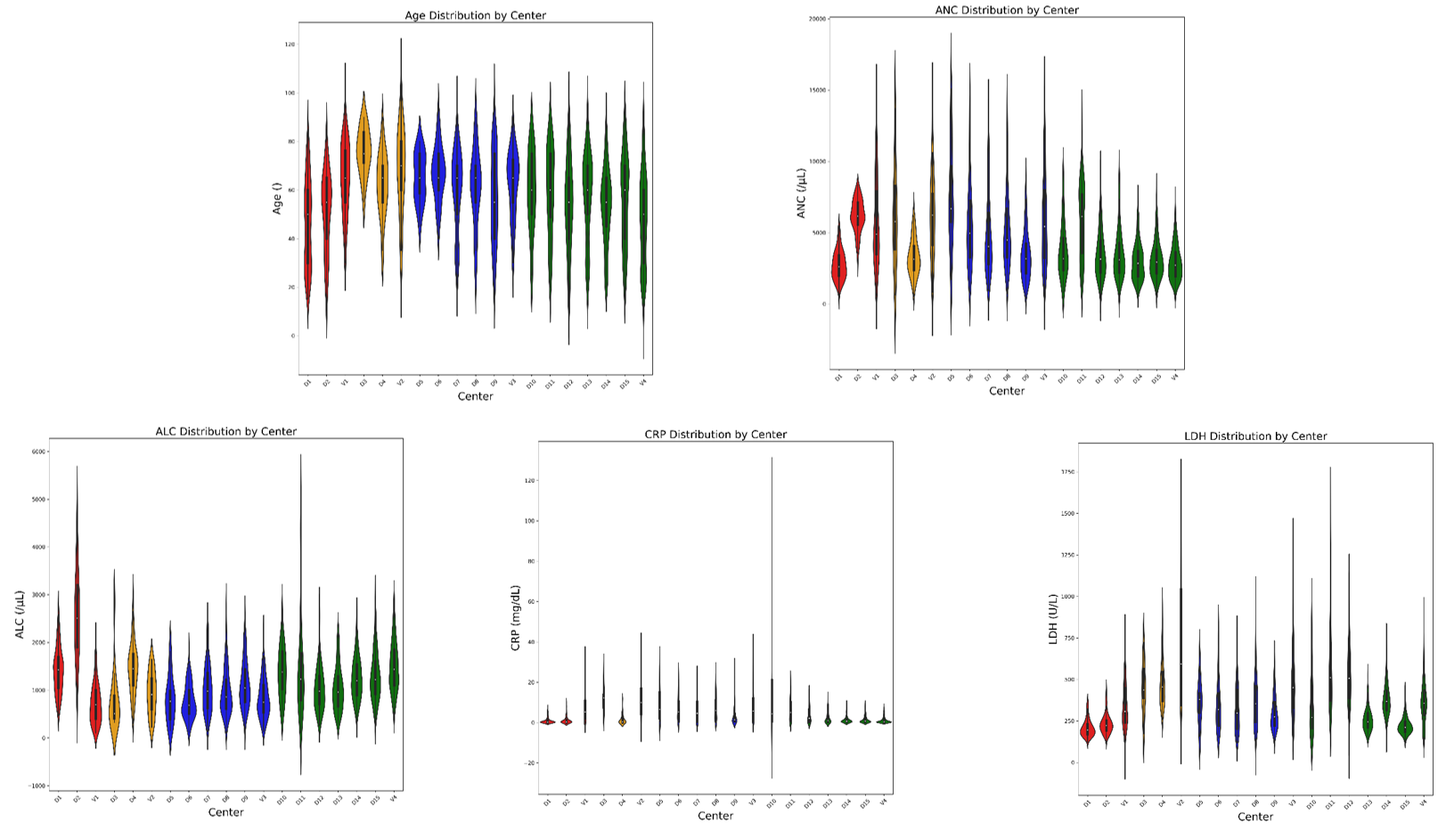


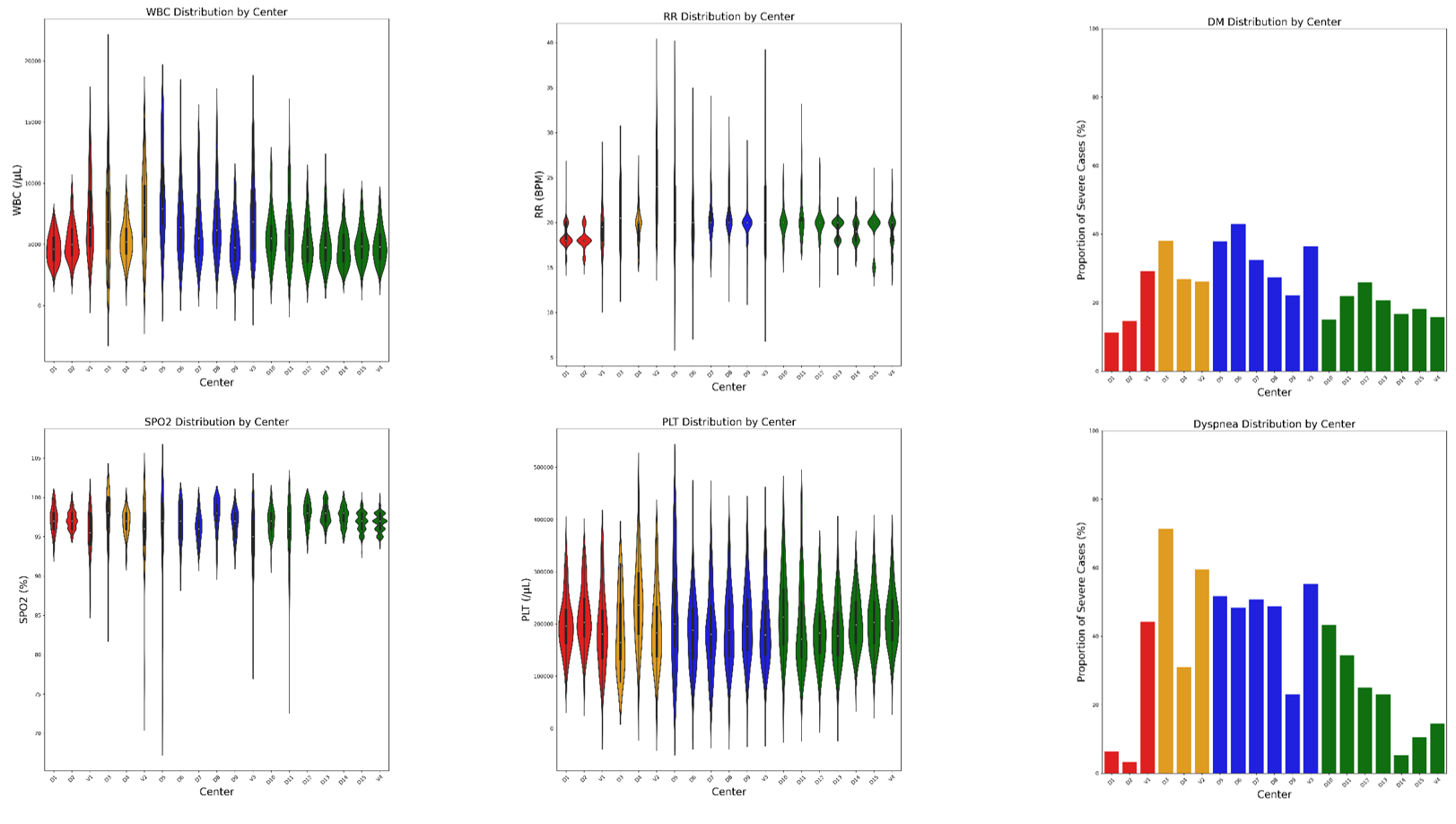
Z-score less than or equal to 3 were included to remove outliers. RIETS=Robust and Interpretable Early Triaging System. ANC=absolute neutrophil count. ALC=absolute lymphocyte count. CRP=c-reactive protein. LDH=lactate dehydrogenase. WBC=white blood cell. SPO2=saturation of peripheral oxygen. RR=respiratory rate. PLT=platelet count.

**Supplementary Figure S7. Empirical distributions of AUROC scores in internal and external validations**


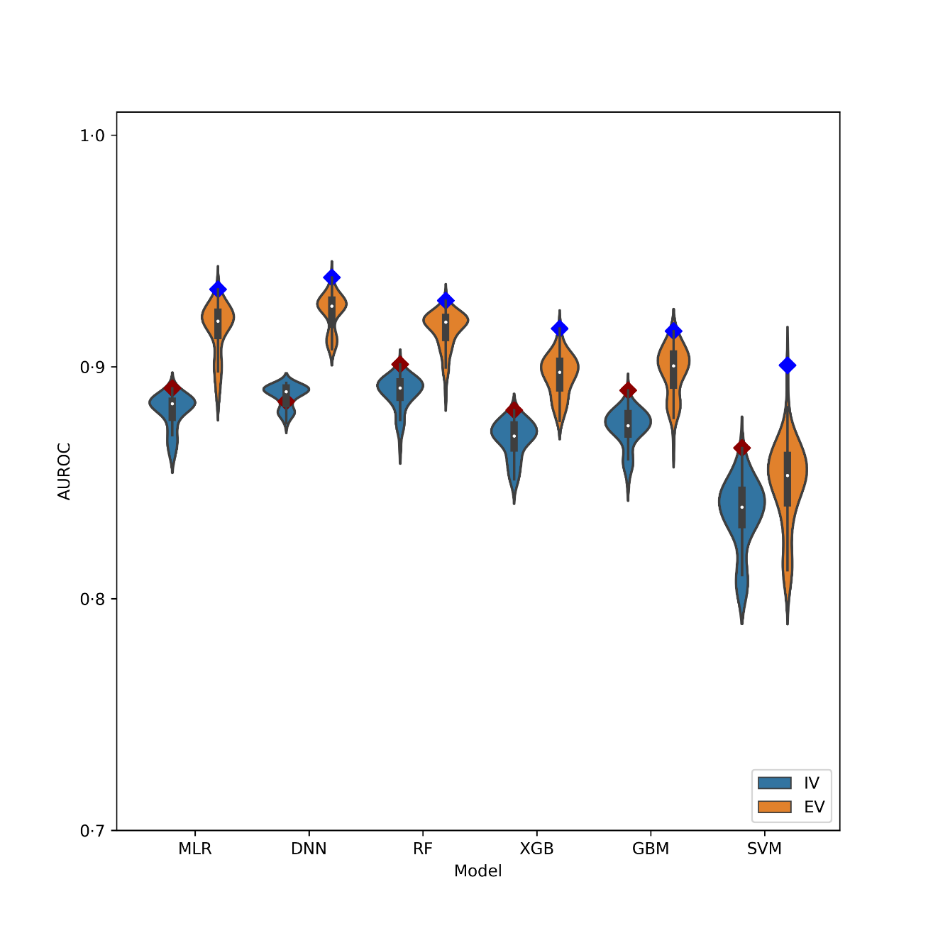


Empirical distributions of area under receiver operating characteristic curve (AUROC) scores were estimated for each prediction model with all 60 feature subsets based on the resamples produced during the internal (IV) and external validations (EV). MLR=multivariable logistic regression. DNN=deep neural network. RF=random forests. XGB=eXtreme gradient boosting. GBM=gradient boosting machine. SVM=support vector machine.

**Supplementary Methods**

**(S1) Study setting and design**

***Source of Data***

The data sets were collected from 19 main referral hospitals across the nation: Ajou University Hospital, Chonnam National University Hospital, Chungang University Hospital, Chungbuk National University Hospital, Chungnam National University Hospital, Chungnam National University Sejong Hospital, Daegu Catholic University Medical Center, Gachon University Gil Medical Center, Jeonbuk National University Hospital, Kangwon National University Hospital, Keimyung University Dongsan Medical Center, Kyungpook National University Hospital, Pusan National University Hospital, Seoul Metropolitan Government Seoul National University Boramae Medical Center, Seoul National University Bundang Hospital, Samsung Medical Center, Seoul National University Hospital, Soonchunhyang University Hospital, and Uijeongbu Eulji Medical Center.

***Data Collection***

The clinical data included demographics (age and sex), comorbidities (hypertension, diabetes, cardiovascular disease, cancer, other), symptoms (fever, cough, sputum, myalgia, dyspnea, sore throat, loss of sensor, gastrointestinal symptom), and vital signs (body temperature, systolic blood pressure, diastolic blood pressure, pulse rate, respiratory rate, saturation pulse oxygen). The laboratory data included white blood cell counts, absolute lymphocyte counts, absolute neutrophil counts, platelet counts, C-reactive protein, and lactate dehydrogenase. Lastly, real-time polymerase chain reaction results were labeled either positive or negative using a cycle threshold value of 40 as the cut-off.

**(S2) Model Development and Validation**

***Six ML-based algorithms***

We chose 6 representative ones from major streams of ML algorithms. The reason was two-fold. First, we wanted to identify robust features against the choice of ML algorithms. Second, the chance to find an optimal ML algorithm with very good performance would be increased by comprehensive search. The chosen ML algorithms included (1) DNN among neural net-based algorithms; (2) RF among bagging-based tree algorithms; (3) XGB, GBM among boosting-based tree algorithms; (4) SVM among margin-based algorithms; and (5) MLR among linear model-based algorithms. If needed, hyperparameters of prediction models were finely tuned by a Bayesian optimization technique.

***RIETS Model Structure***

RIETS is a DNN-based final prediction model. Its hyperparameters were empirically tuned and we achieved the best performance with the following parameters: a batch size of 32, an epoch of 50, an early stopper with patience level of 5, an Adam optimizer with learning rate of 0.01, a ReduceLROnPlateau learning rate scheduler—reduces when validation loss stops improving—with patience level of 1 and factor of 0.8, an input layer with the size of 128, two hidden layers with the size of 32 and 16, an output layer with sigmoid activation layer. Between hidden layers, the model was constructed with ReLU activation layer followed by a dropout layer with a 0.3 ratio and a batch normalization layer.

***Random States for Cross-Validation and Bootstrapping***

Different random states were used to partition the development cohort iteratively, and to obtain 100 bootstrap samples from the (external) validation cohort. In the meanwhile, random state parameters of all ML algorithms were fixed to a specific value (i.e., 0) that the randomness introduced in the algorithm was consistent across different trials.

***95% Confidence Intervals of Performance Metrics***

First, we estimated the values of each performance metric based on the five iterations of stratified five-fold cross-validation and the 100 bootstrap samples for internal and external validations, respectively. For each performance metric, we used these estimated values to generate the empirical distribution by assuming the normality with sample mean and standard deviation. Finally, 95% confidence intervals were estimated from the empirical distribution.

**(S3) Model Performance Visualization and Interpretation**

***Calibration Plots^1^***

The external cohort were segmented into 10 equal-sized bins according to the predicted probability for patients. For each bin, we calculated the observed risk as the ratio of the severe patients, and the predicted risk as the mean of predicted probabilities for all patients in the bin. The scatter plot of predicted and observed risks was constructed with 95% confidence bands for the predicted risk and the reference dashed line of identity function. After all, integrated calibration index (ICI) was derived from the weighted average of the absolute difference between the calibration curve and the perfectly calibrated diagonal line.^2^

***SHAP Summary Plots^3^***

SHAP summary plot, a tool derived from cooperative game theory, quantifies the contribution of each feature towards the prediction outcome. Shapley values are calculated based on the marginal contribution of each feature to the prediction while considering all possible combinations of features. These values are then plotted to a summary plot for a visual representation of the importance of each feature. Since SHAP values are not conventionally computed with DNN-based models, we properly modified the KernelExplainer method appropriately to compute SHAP values for features in RIETS

***Decision Curve Analysis Plots^4^***

Using each candidate threshold value of the predicted probability, we divided the patients into low- and high-risk groups. Then we assessed the potential net benefit of this risk grouping when applied to clinical decisions on intervention (i.e., intervention only for patients with high risk). The potential net benefit for this model-based intervention strategy was displayed across threshold values, along with two references, such as ‘always treat (i.e., intervention for all)’ and ‘never treat (i.e., intervention for none).’ We applied locally weighted smoothing to smooth the decision curves.

***DDRTree Plots^5^***

To construct DDRTree, we first preprocessed our dataset by removing outliers from our patient cohort (4,787 patients; 80.5% remained) based on 2% upper and lower thresholds following the logarithmic transformation to each continuous feature. Then, we tuned the hyperparameters (sigma 0.03 and gamma 0.007) through GridSearch and applied alpha shading (0.3) and jitter (0.04) to illuminate the high density regions. Lastly, we computed the residuals of each laboratory feature adjusted for the preexisting features (age, DM, and dyspnea) using multiple linear regression model.

**(S4) Model Transportability to New Variant Dominant Period**

***Definition of Variant-Dominant Periods***

The predominant circulating variant at the time of hospitalization was identified through viral whole genome sequencing, and could differ across nations.^6^ According to predominant circulating variants during the pandemic in South Korea, we segmented our study periods into three variant-dominant periods, and constructed the corresponding patient sub-cohorts: Original-Alpha dominant period (January 5^th^, 2020 ~ May 1^st^, 2021), Delta dominant period (May 1^st^, 2021 ~ November 24^th^, 2021), and Omicron dominant period (November 24^th^, 2021 ~ August 24^th^, 2022).^6^

**Analysis of Model Transportability on Omicron variant cases**

We developed modifications of RIETS to explore its prediction transportability across different variant dominant periods. Each modified model was constructed using the variant dominant sub-cohorts in the development cohort. For instance, ‘RIETS-All’ model was based on entire development cohort and ‘RIETS-Omicron’ model was based on Omicron dominant development cohort. We evaluated all possible combinations of modified RIETS and compared their discriminative performances among patients in external validation cohort. Consequently, we identified best-performing model named ‘RIETS-Ensemble’ that integrates the three models based on Original-Alpha-Omicron, Delta-Omicron, and Omicron. Then, ‘RIETS-Ensemble’ model was contrasted to ‘RIETS-All’ and ‘RIETS-Omicron’ models to visualize marginal improvements. All developed models were compared by using AUROC as a measure for discriminative performance.

**(S5) Statistical analysis**

All statistical analyses were performed using Python (Python Software Foundation, version 3·9) with the following libraries: ‘Pandas’ for dataset organization and cleansing; ‘Hyperopt’^7^ for hyperparameter tuning; ‘Sci-kit Learn’ for data normalization, loading ML algorithms (MLR, RF, SVM, GBM), feature importance and predictive metrics computation, and stratified cross validation; ‘Xgboost’ for XGB model load; ‘Tensorflow’ for DNN model development; ‘SHAP’ for candidate features generation and individual feature interpretation; ‘Matplotlib’ for model result visualization (ROC curve analyses, calibration plot, decision curve analysis plot, and reclassification plot); ‘Seaborn’ for violin and bar plots. ‘dynamo’ for DDR Tree generation.

**Supplementary File S1: TRIPOD Checklist for Prediction Model Development and Validation**

**
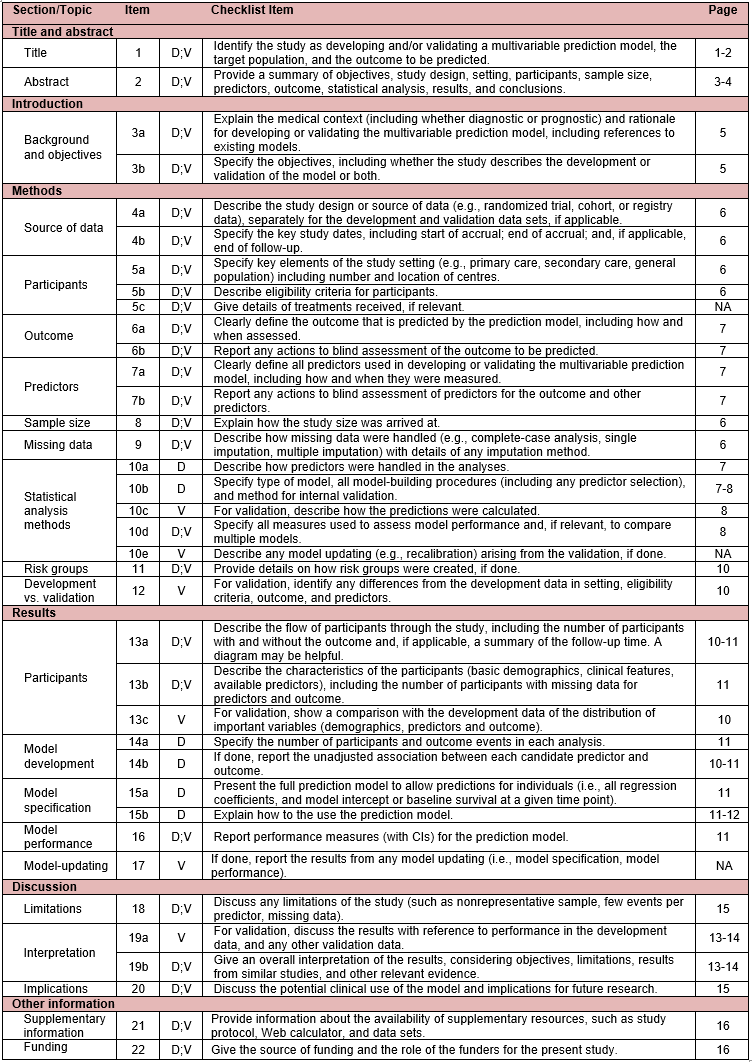
**

*Items relevant only to the development of a prediction model are denoted by D, items relating solely to a validation of a prediction model are denoted by V, and items relating to both are denoted D;V. We recommend using the TRIPOD Checklist in conjunction with the TRIPOD Explanation and Elaboration document.

Supplementary File S2: PROBAST Guideline for Prediction model study Risk Of Bias Assessment Tool

Published in Annals of Internal Medicine (freely available):

1. [PROBAST: A Tool to Assess the Risk of Bias and Applicability of Prediction Model Studies](https://annals.org/aim/fullarticle/2719961/probast-tool-assess-risk-bias-applicability-prediction-model-studies)
2. [PROBAST: A Tool to Assess Risk of Bias and Applicability of Prediction Model Studies: Explanation](https://annals.org/aim/fullarticle/2719962/probast-tool-assess-risk-bias-applicability-prediction-model-studies-explanation) [and Elaboration](https://annals.org/aim/fullarticle/2719962/probast-tool-assess-risk-bias-applicability-prediction-model-studies-explanation)

# What does PROBAST assess?

PROBAST assesses both the *risk of bias* and *concerns regarding applicability* of a study that evaluates (develops, validates or updates) a multivariable diagnostic or prognostic prediction model. It is designed to assess primary studies included in a systematic review.

*Bias* occurs if systematic flaws or limitations in the design, conduct or analysis of a primary study distort the results. For the purpose of prediction modelling studies, we have defined *risk of bias* to occur when shortcomings in the study design, conduct or analysis led to systematically distorted estimates of a model’s predictive performance or to an inadequate model to address the research question. Model predictive performance is typically evaluated using calibration, discrimination and sometimes classification measures, and these are likely inaccurately estimated in studies with high risk of bias. *Applicability* refers to the extent to which the prediction model from the primary study matches your systematic review question, for example in terms of the participants, predictors, or outcome of interest.

A primary study may include the development and/or validation or update of more than one prediction model. A PROBAST assessment should be completed for each distinct model that is developed, validated or updated (extended) for making individualized predictions. Where a publication assesses multiple prediction models, only complete a PROBAST assessment for those models that meet the inclusion criteria for your systematic review. Please note that subsequent use of the term “model” includes derivatives of models, such as simplified risk scores, nomograms, or recalibrations of models.

PROBAST is not designed for all multivariable diagnostic or prognostic studies. For example, studies using multivariable models to identify predictors associated with an outcome but not attempting to develop a model for making individualized predictions are not covered by PROBAST.

PROBAST includes four steps.

| **Step** | **Task** | **When to complete** |
| --- | --- | --- |
| **1** | Specify your systematic review question(s) | Once per systematic review |
| **2** | Classify the type of prediction model  evaluation | Once for each model of interest in each publication  being assessed, for each relevant outcome |
| **3** | Assess risk of bias and applicability | Once for each development and validation of each distinct prediction model in a publication |
| **4** | Overall judgment | Once for each development and validation of each  distinct prediction model in a publication |

If this is your first time using PROBAST, we strongly recommend reading the detailed explanation and elaboration (E&E, see link above) paper and to check the examples on [www.probast.org](http://www.probast.org/)

# Step 1: Specify your systematic review question.

State your systematic review question to facilitate the assessment of the applicability of the evaluated models to your question. *The following table should be completed once per systematic review.*

| **Criteria** | **Specify your systematic review question** |
| --- | --- |
| *Intended use of model:* | To support clinical decision-making during early triaging for hospitalized COVID-19 patients |
| ***Participants*** *including selection criteria and setting:* | *Adult COVID-19 patients confirmed by RT-PCR test* |
| ***Predictors*** *(used in prediction modelling), including types of predictors (e.g. history, clinical examination, biochemical markers, imaging tests), time of measurement, specific measurement issues (e.g., any requirements/ prohibitions for specialized*  *equipment):* | History, clinical examination, biochemical markers were the predictors used for the study.  All predictors were collected within the first day of hospitalization. For complete-case analysis, patients with missing data and patients diagnosed either more than 15 days before or more than 1 day after the hospitalization were excluded. |
| *Outcome to be predicted:* | *Severe cases defined according to clinical progression scale for COVID-19* |

# Step 2: Classify the type of prediction model evaluation.

Use the following table to classify the evaluation as model development, model validation or model update, or combination. Different signaling questions apply for different types of prediction model evaluation. If the evaluation does not fit one of these classifications, then PROBAST should not be used.

| **Classify the evaluation based on its aim** | | | |
| --- | --- | --- | --- |
| **Type of**  **prediction study** | **PROBAST boxes**  **to complete** | **Tick as**  **appropriate** | **Definition for type of prediction model study** |
| Development only | Development |  | Prediction model development without external validation. These studies may include internal validation methods, such as bootstrapping and  cross-validation techniques. |
| Development and validation | Development and validation | O | Prediction model development combined with external validation in other participants in the same  article. |
| Validation only | Validation |  | External validation of existing (previously  developed) model in other participants. |

| *This table should be completed once for each publication being assessed and for each relevant outcome in*  *your review.* | |
| --- | --- |
| **Publication reference** | Prediction models for diagnosis and prognosis of COVID-19: systematic review and critical appraisal |
| **Models of interest** | COVID-19 Patients Prognosis Prediction |
| **Outcome of interest** | COVID-19 Severity |

# Step 3: Assess risk of bias and applicability

PROBAST is structured as four key domains. Each domain is judged for risk of bias (low, high or unclear) and includes signaling questions to help make judgements. Signaling questions are rated as yes (Y), probably yes (PY), probably no (PN), no (N) or no information (NI). All signaling questions are phrased so that “yes” indicates absence of bias. Any signaling question rated as “no” or “probably no” flags the potential for bias; you will need to use your judgement to determine whether the domain should be rated as “high”, “low” or “unclear” risk of bias. The guidance document contains further instructions and examples on rating signaling questions and risk of bias for each domain.

The first three domains are also rated for concerns regarding applicability (low/ high/ unclear) to your review question defined above.

*Complete all domains separately for each evaluation of a distinct model. Shaded boxes indicate where signaling questions do not apply and should not be answered.*

| **DOMAIN 1: Participants** | | | |
| --- | --- | --- | --- |
| **A. Risk of Bias** | | | |
| *Describe the sources of data and criteria for participant selection:*  A nationwide 19 main referral hospitals in South Korea who were confirmed to have COVID-19  via RT-PCR | | | |
|  | | Dev | Val |
| 1.1 Were appropriate data sources used, e.g. cohort, RCT or nested case-control study  data? | | Y | Y |
| 1.2 Were all inclusions and exclusions of participants appropriate? | | Y | Y |
| **Risk of bias introduced by selection of participants** | **RISK:**  *(low/ high/ unclear)* | **Low** | **Low** |
| *Rationale of bias rating:*  We collected patient cohort from 19 hospitals, all inclusion and exclusion criterion were appropriate. | | | |
| **B. Applicability** | | | |
| *Describe included participants, setting and dates:*  Participants were collected from 19 main referral hospitals in South Korea between January 5th 2020 and August 29th 2022. | | | |
| **Concern that the included participants and setting do not match**  **the review question** | **CONCERN:**  *(low/ high/ unclear)* | **Low** | **Low** |
| *Rationale of applicability rating:*  Our cohort satisfies applicability because the cohort was constructed using multi-centers and prospectively collected for three years. | | | |

| **DOMAIN 2: Predictors** | | | |
| --- | --- | --- | --- |
| **A. Risk of Bias** | | | |
| *List and describe predictors included in the final model, e.g. definition and timing of assessment:*  lactate dehydrogenase, age, absolute lymphocyte counts, dyspnea, respiratory rate, diabetes mellitus, c-reactive protein, absolute neutrophil counts, platelet counts, white blood cell counts, saturation of peripheral oxygen were predictors included in the final model. We collected these predictors within first day of hospitalization | | | |
|  | | Dev | Val |
| 2.1 Were predictors defined and assessed in a similar way for all participants? | | Y | Y |
| 2.2 Were predictor assessments made without knowledge of outcome data? | | Y | Y |
| 2.3 Are all predictors available at the time the model is intended to be used? | | Y | Y |
| **Risk of bias introduced by predictors or their assessment** | **RISK:**  *(low/ high/ unclear)* | **Low** | **Low** |
| *Rationale of bias rating:*  All selected predictors satisfy the conditions listed above: 1) defined and assessed in a similar way for all participants, assessments made without knowledge of outcome data, available at the time of intended use | | | |
| **B. Applicability** | | | |
| Concern that the definition, assessment or timing of predictors in  the model do not match the review question | **CONCERN:**  *(low/ high/ unclear)* | **Low** | **Low** |
| *Rationale of applicability rating:*  All predictors used in the model were routinely collected features that they are applicable at wide range of clinical settings. | | | |

| **DOMAIN 3: Outcome** | | | |
| --- | --- | --- | --- |
| **A. Risk of Bias** | | | |
| *Describe the outcome, how it was defined and determined, and the time interval between predictor assessment and outcome determination:*  COVID-19 severity was defined based on patients fulfilling one or more of the following conditions during their hospitalization:  1) patients requiring mechanical ventilation  2) patients requiring extracorporeal membrane oxygenation;  3) patients admitted to intensive care unit  4) patients who passed away.  This outcome was defined in accordance with clinical progression scale for COVID-19. Time interval between predictor assessment and outcome determination was 15 days since the purpose of this research was the short-term patient prognosis prediction. | | | |
|  | | Dev | Val |
| 3.1 Was the outcome determined appropriately? | | Y | Y |
| 3.2 Was a pre-specified or standard outcome definition used? | | Y | Y |
| 3.3 Were predictors excluded from the outcome definition? | | Y | Y |
| 3.4 Was the outcome defined and determined in a similar way for all participants? | | Y | Y |
| 3.5 Was the outcome determined without knowledge of predictor information? | | Y | Y |
| 3.6 Was the time interval between predictor assessment and outcome determination  appropriate? | | Y | Y |
| **Risk of bias introduced by the outcome or its determination** | **RISK:**  *(low/ high/ unclear)* | **Low** | **Low** |
| *Rationale of bias rating:*  The outcome was determined appropriately, followed standard outcome definition set by WHO, predictors were excluded from outcome, outcome was defined and determined similarly for all participants, time interval between predictor assessment and outcome determination was appropriate | | | |
| **B. Applicability** | | | |
| *At what time point was the outcome determined:*  Outcome was determined after 15 days from hospitalization  *If a composite outcome was used, describe the relative frequency/distribution of each contributing outcome:*  Composite outcome was not used. | | | |
| **Concern that the outcome, its definition, timing or**  **determination do not match the review question** | **CONCERN:**  *(low/ high/ unclear)* | **Low** | **Low** |
| *Rationale of applicability rating:*  The time point of outcome determination was relevant in the scope of this study, so the applicability satisfies. | | | |

| **DOMAIN 4: Analysis** | | | |
| --- | --- | --- | --- |
| **Risk of Bias** | | | |
| *Describe numbers of participants, number of candidate predictors, outcome events and events per candidate predictor:*  *5,945 adult patients were enrolled in the study. We used total 27 candidate predictors. Outcome events were either non-severe or severe COVID. Distribution of events per candidate predictor is in Supplementary Fig. 2.* | | | |
| *Describe how the model was developed (for example in regards to modelling technique (e.g. survival or logistic modelling), predictor selection, and risk group definition):*  We first identified 60 candidate feature subsets using stably-selected features generated by feature engineering methods. Then, we simultaneously developed and assessed the predictive performance of all potential combinations to find best prediction model (see Fig. 2 for details). | | | |
| *Describe whether and how the model was validated, either internally (e.g. bootstrapping, cross validation, random split sample) or externally (e.g. temporal validation, geographical validation, different setting, different type of participants):*  *The model was validated using stratified 5-fold cross validation during internal validation. After, we bootstrapped the external validation cohorts comprised of different temporal period and geograpihcal location to externally validate the model* | | | |
| *Describe the performance measures of the model, e.g. (re)calibration, discrimination, (re)classification, net benefit, and whether they were adjusted for optimism:*  *The model was evaluated through calibration plot, reclassification plot, and decision curve analysis plot. The*  *model demonstrated the exceptional performance in all measures, for those adjustments was not necessary* | | | |
| *Describe any participants who were excluded from the analysis:*  *Patients with missing data and diagnosed either more than 15 days before or more than 1 day after*  *hospitalization was excluded from the analysis.* | | | |
| *Describe missing data on predictors and outcomes as well as methods used for missing data:*  *There was no missing data because we conducted complete-case analysis. We excluded all missing data* | | | |
|  | | Dev | Val |
| 4.1 Were there a reasonable number of participants with the outcome? | | Y | Y |
| 4.2 Were continuous and categorical predictors handled appropriately? | | Y | Y |
| 4.3 Were all enrolled participants included in the analysis? | | Y | Y |
| 4.4 Were participants with missing data handled appropriately? | | Y | Y |
| 4.5 Was selection of predictors based on univariable analysis avoided? | | Y | Y |
| 4.6 Were complexities in the data (e.g. censoring, competing risks, sampling of controls)  accounted for appropriately? | | Y | Y |
| 4.7 Were relevant model performance measures evaluated appropriately? | | Y | Y |
| 4.8 Were model overfitting and optimism in model performance accounted for? | | Y | Y |
| 4.9 Do predictors and their assigned weights in the final model correspond to the results  from multivariable analysis? | | Y | Y |
| **Risk of bias introduced by the analysis** | **RISK:**  *(low/ high/ unclear)* | **Low** | **Low** |
| *Rationale of bias rating:*  Reasonal number of patients with outcome were included for the analysis. The predictors were handled properly. Complete-case analysis was conducted. Exhaustively searched during predictor and algorithm selection that optimal model is discovered and overfitting was avoided. Complexities in the data were accounted appropriately, all relevant performance measures were performed. | | | |

# Step 4: Overall assessment

Use the following tables to reach overall judgements about risk of bias and concerns regarding applicability of the prediction model evaluation (development and/or validation) across all assessed domains.

*Complete for each evaluation of a distinct model.*

| **Reaching an overall judgement about risk of bias of the prediction model evaluation** | |
| --- | --- |
| **Low risk of bias** | If all domains were rated low risk of bias.  If a prediction model was developed without any external validation, and it was rated as low risk of bias for all domains, consider downgrading to **high risk of bias**. Such a model can only be considered as low risk of bias, if the development was based on a very large data set and included some form of internal validation. |
| **High risk of bias** | If at least one domain is judged to be at **high risk of bias**. |
| **Unclear risk of**  **bias** | If an unclear risk of bias was noted in at least one domain and it was low risk for all  other domains. |

| **Reaching an overall judgement about applicability of the prediction model evaluation** | |
| --- | --- |
| **Low concerns regarding**  **applicability** | If low concerns regarding applicability for all domains, the prediction model  evaluation is judged to have **low concerns regarding applicability**. |
| **High concerns regarding**  **applicability** | If high concerns regarding applicability for at least one domain, the prediction  model evaluation is judged to have **high concerns regarding applicability**. |
| **Unclear concerns regarding applicability** | If unclear concerns (but no “high concern”) regarding applicability for at least  one domain, the prediction model evaluation is judged to have **unclear concerns regarding applicability** overall. |

| **Overall judgement about risk of bias and applicability of the prediction model evaluation** | | |
| --- | --- | --- |
| **Overall judgement of risk of bias** | **RISK:**  *(low/ high/ unclear)* | **Low** |
| *Summary of sources of potential bias:*  The prediction model rated low risk for all domains listed in this evaluation criterion. | | |
| **Overall judgement of applicability** | **CONCERN:**  *(low/ high/ unclear)* | **Low** |
| *Summary of applicability concerns:*  The prediction model rated low risk for all domains listed in this evaluation criterion. | | |

**Supplementary References**

1. Van Calster B, McLernon DJ, van Smeden M, et al. Calibration: the Achilles heel of predictive analytics. *BMC Med*. Dec 16 2019;17(1):230. doi:10.1186/s12916-019-1466-7

2. Austin PC, Steyerberg EW. The Integrated Calibration Index (ICI) and related metrics for quantifying the calibration of logistic regression models. *Stat Med*. Sep 20 2019;38(21):4051-4065. doi:10.1002/sim.8281

3. Jia R, Dao D, Wang B, et al. Towards Efficient Data Valuation Based on the Shapley Value. presented at: Proceedings of the Twenty-Second International Conference on Artificial Intelligence and Statistics; 2019; Proceedings of Machine Learning Research. <https://proceedings.mlr.press/v89/jia19a.html>

4. Vickers AJ, van Calster B, Steyerberg EW. A simple, step-by-step guide to interpreting decision curve analysis. *Diagn Progn Res*. 2019;3:18. doi:10.1186/s41512-019-0064-7

5. Qiu X, Mao Q, Tang Y, et al. Reversed graph embedding resolves complex single-cell trajectories. *Nat Methods*. Oct 2017;14(10):979-982. doi:10.1038/nmeth.4402

6. Lee DW, Kim JM, Park AK, et al. Genomic epidemiology of SARS- CoV-2 Omicron variants in the Republic of Korea. *Sci Rep*. Dec 27 2022;12(1):22414. doi:10.1038/s41598-022-26803-w

7. Bergstra J, Komer B, Eliasmith C, Yamins D, Cox DD. Hyperopt: a Python library for model selection and hyperparameter optimization. *Computational Science & Discovery*. 2015;8(1)doi:10.1088/1749-4699/8/1/014008
